# Uncovering key clinical trial features influencing recruitment

**DOI:** 10.1017/cts.2023.623

**Published:** 2023-09-04

**Authors:** Betina Idnay, Yilu Fang, Alex Butler, Joyce Moran, Ziran Li, Junghwan Lee, Casey Ta, Cong Liu, Chi Yuan, Huanyao Chen, Edward Stanley, George Hripcsak, Elaine Larson, Karen Marder, Wendy Chung, Brenda Ruotolo, Chunhua Weng

**Affiliations:** 1 Department of Biomedical Informatics, Columbia University Irving Medical Center, New York, NY, USA; 2 Department of Neurology, Columbia University Irving Medical Center, NY Research, New York, NY, USA; 3 Compliance Applications, Information Technology, Columbia University, New York, NY, USA; 4 School of Nursing, Columbia University Irving Medical Center, New York, NY, USA; 5 New York Academy of Medicine, New York, NY, USA; 6 Department of Pediatrics, Columbia University Irving Medical Center, New York, NY, USA; 7 Institutional Review Board for Human Subjects Research, Columbia University, New York, NY, USA

**Keywords:** Clinical trials, research recruitment, machine learning, SHAP, informatics

## Abstract

**Background:**

Randomized clinical trials (RCT) are the foundation for medical advances, but participant recruitment remains a persistent barrier to their success. This retrospective data analysis aims to (1) identify clinical trial features associated with successful participant recruitment measured by accrual percentage and (2) compare the characteristics of the RCTs by assessing the most and least successful recruitment, which are indicated by varying thresholds of accrual percentage such as ≥ 90% vs ≤ 10%, ≥ 80% vs ≤ 20%, and ≥ 70% vs ≤ 30%.

**Methods:**

Data from the internal research registry at Columbia University Irving Medical Center and Aggregated Analysis of ClinicalTrials.gov were collected for 393 randomized interventional treatment studies closed to further enrollment. We compared two regularized linear regression and six tree-based machine learning models for accrual percentage (i.e., reported accrual to date divided by the target accrual) prediction. The outperforming model and Tree SHapley Additive exPlanations were used for feature importance analysis for participant recruitment. The identified features were compared between the two subgroups.

**Results:**

CatBoost regressor outperformed the others. Key features positively associated with recruitment success, as measured by accrual percentage, include government funding and compensation. Meanwhile, cancer research and non-conventional recruitment methods (e.g., websites) are negatively associated with recruitment success. Statistically significant subgroup differences (corrected *p*-value < .05) were found in 15 of the top 30 most important features.

**Conclusion:**

This multi-source retrospective study highlighted key features influencing RCT participant recruitment, offering actionable steps for improvement, including flexible recruitment infrastructure and appropriate participant compensation.

## Introduction

Randomized clinical trials (RCTs) have long been the gold standard for generating high-quality medical evidence [1]. The success of RCTs depends on the timely accrual of a representative and qualified study sample, but this remains a challenge [1,2]. Fewer than 4% of adults in the United States (US) participate in clinical trials [2– 4], and this number has not improved since 1994, despite increasingly prolonged recruitment periods [5,6]. Further, up to 85% of clinical trials fail to recruit or retain a sufficient sample size, leading to failures to meet accrual targets in four out of every five trials, even though nearly $1.9 billion is spent on recruitment annually [2]. Moreover, the lack of diversity and representativeness in study populations is another persistent problem. All of these cause study delays, increase costs, limit statistical power, and subsequently compromise clinical trial quality [7]. It is imperative to develop methods to optimize the trial design for better feasibility, inclusiveness, and recruitment efficiency to improve the sustainability and impact of clinical trial research.

Several studies have assessed the impact of individual clinical trial characteristics on recruitment success [8–10]. Factors contributing to successful recruitment include funding type (e.g., a federal agency, pharmaceutical company), trial phase (phase II having faster recruitment than phase I or phase III trials), and type of trial site (research facility or other) [11,12]. Other studies have focused on the role of the clinician or the patient in trial recruitment. Clinician efforts toward administrative preparation of the study site, increasing public awareness, and trial recommendations have enhanced enrollment, while the effectiveness of particular recruitment methods remains unclear [13,14]. Patient factors, including insurance coverage (or lack of), perceived drawbacks of participating in research, time and travel constraints, and perception of therapeutic benefit, have been shown to directly impact the likelihood of patient enrollment [12]. A potential limitation in these studies is that many focused on a specific disease domain (e.g., oncology) or patient population (e.g., pediatrics), limiting the generalizability of the findings [8–10].

This study extends prior work to systematically identify clinical trial features associated with recruitment success by employing large database analyses using linked clinical trial registries (one nationally managed and one at a single facility). In this study, we measured RCT recruitment success by accrual percentage [1,2]. Two regularized linear regression and six tree-based machine learning algorithms were compared, and the optimal algorithm (i.e., CatBoost [15] regressor) was applied to predict the accrual percentage of RCTs. While interpretability has been considered critical in the domain, existing works lack a comprehensive analysis of feature importance. In this work, we used Tree SHapley Additive exPlanations (SHAP) [16] for a detailed analysis of feature importance for participant recruitment to RCTs. We further conducted a subgroup analysis between RCTs with high and low accrual percentages indicated by three sets of thresholds, including ≥ 90% and ≤ 10%, ≥ 80% and ≤ 20%, as well as≥70% and ≤ 30%. Finally, recommendations for engaging stakeholders to improve recruitment are provided.

## Materials and Methods

**Figure 1.**
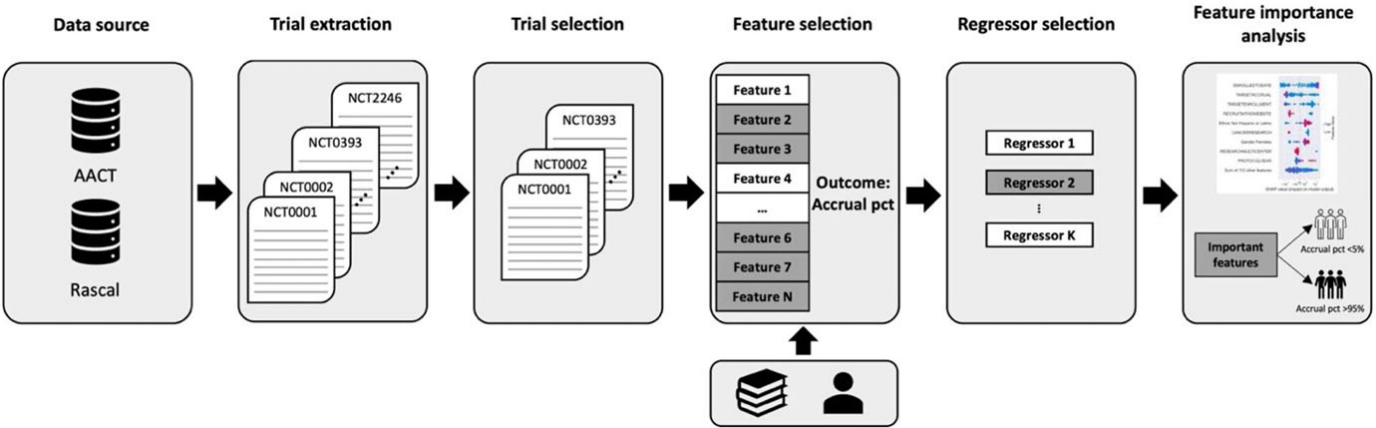
Overall study methodology.

We conducted a multi-source retrospective data analysis using machine learning methods to investigate the impact of evidence-based and expert-identified features on the success of RCT recruitment. Fig. 1 depicts the overall methodology and databases used in this study.

### Data Source and Trial Selection

We used two data sources: (1) Research Compliance and Administration System (RASCAL, https://rascal.columbia.edu/), a single-institution electronic clinical research registry; and (2) the Aggregate Analysis of ClinicalTrials.gov (AACT) database, a global clinical trials database by the US National Library of Medicine (https://clinicaltrials.gov/) [17]. We extracted clinical trials from RASCAL with protocol approval dates ranging from 06/04/2015 to 07/31/2019. We included randomized interventional treatment studies that were closed to further enrollment. Studies with multiple registered protocols in RASCAL or were terminated due to non-recruitment-related reasons such as loss of funding, study drug toxicity, or other administrative reasons were excluded. Additional recruitment details (i.e., number of study sites and target domain) were extracted from the AACT. Finally, studies without reported target accrual (*n* = 47) or with greater than 100% accrual percentage in RASCAL (*n* = 7) were excluded from the main analysis. Accruing more than the approved number of subjects is a violation per Columbia University Irving Medical Center’s Institutional Review Board (IRB). Though the IRB assessed studies with reported over-accrual, we cannot be certain if the information was mistakenly reported (i.e., typographical error) or if it was deemed a violation, hence the exclusion.

### Data Processing and Feature Selection

We selected features based on a combination of evidence in the literature (e.g., recruitment methods [18], resources for research staff [19,20] study design [21–23], randomization [24,25], and consent process [21]) and domain expertise (BI: 7 years as a research nurse and recruitment coordinator; JM: over 20 years as clinical research staff and eight years as multi-site project manager). A detailed list of the extracted and selected features with the selection rationale is included in Supplementary Table S1.

We distinguished the difference between enrollment and accrual based on the RASCAL definition. Individuals who agree to participate in a study, even if just for screening or assessment purposes, are considered to be enrolled in the study. On the other hand, individuals who are confirmed to be eligible for an interventional study with a screening procedure to determine eligibility that occurs after consent is obtained are regarded as accrual. The accrual-to-date number is a subset of the number of enrolled participants. Our outcome of interest, accrual percentage, was calculated by dividing the reported accrual to date by the target accrual.

For all binary variables, such as the recruitment methods class of features, we assumed that a missing value indicates the absence of a feature. One-hot encoding was applied to polytomous variables (categorical variables with more than two possible values), such as the study phase. The target clinical domain of an RCT was extracted from the relevant Medical Subject Headings (MeSH) terms displayed on ClinicalTrials.gov. MeSH are standardized keywords from a controlled and hierarchically organized vocabulary produced by the National Library of Medicine and are publicly available at http://www.ncbi.nlm.nih.gov/pubmed/advanced. For those without a relevant MeSH term, we manually mapped the conditions of an RCT to MeSH terms. Data processing was described in the Supplementary Material 1.

Finally, we utilized Pearson’s correlation coefficient to quantify the relationship between two continuous variables, the Phi correlation coefficient to evaluate correlations between two dichotomous variables, and the Point-biserial correlation coefficient for examining the association between a continuous variable and a dichotomous one.

### Model Training and Evaluation

To identify factors associated with successful RCT recruitment, we first built a model to predict the accrual percentage with the selected features. We applied and compared two regularized linear regression models (i.e., Ridge regression with l2 regularization [26] and Least Absolute Shrinkage and Selection Operator [Lasso] regression with l1 regularization [27]) and six tree-based machine learning models, including the Decision Tree [28], Random Forest [29], AdaBoost (Adaptive Boosting) [30], XGBoost (extreme gradient boosting algorithm) [31], LightGBM (Light Gradient Boosted Machine) [32], and CatBoost (Categorical Boosting) [15]. We used the Classification and Regression Trees for the Decision Tree regression, which predicts the target by learning decision rules from features [28]. It iteratively splits data into two groups based on the feature that minimizes the cost metric until reaching the stopping criteria. It has a tree-like structure with interior nodes representing features and decision rules and leaf nodes containing a prediction score. Random forest regression combines multiple decision trees, each of which is trained on a bootstrap sample from the dataset and a random subset of features and averages the predictions to control overfitting to yield better performance [29]. AdaBoost regression is a boosting ensemble model that sequentially fits a regressor on the whole dataset with adjusted weights determined by the errors in the current prediction [30]. Decision Tree was selected as the regressor in this model in our study. XGBoost, a more robust gradient-boosted trees algorithm with a regularized objective function, iteratively adds decision trees built by learning the errors in prior trees [31]. LightGBM is also a gradient-boosting algorithm with Gradient-based One-Side Sampling and Exclusive Feature Bundling to achieve better efficiency and scalability [32]. CatBoost, another gradient boosting method, introduces ordered boosting and an algorithm for categorical features to solve the prediction shift issue [15].

We tuned each model’s parameters (Supplementary Table S2) by using 50-times repeated 10-fold cross-validation with grid search. In our effort to mitigate overfitting, we closely monitored the disparity between the mean Root Mean Square Error (RMSE) for the training and validation sets. The optimal parameter configuration was found based on the lowest mean validation RMSE. Subsequently, employing this optimally tuned parameter setting, we trained the model on the entirety of the dataset to inform the subsequent analysis. We also investigated how consistently the top three best-performing models identified the important features, thereby adding more confidence to the interpretation.

Moreover, for comprehensiveness of our analysis, we conducted a supplemental analysis that incorporated the seven studies excluded due to having an accrual percentage greater than 100% in RASCAL, despite the limitation that we cannot tell if these studies represented typographical errors or actual IRB violations, by using the best-performing model to explore the potential impact on our results.

### Feature Importance Analysis

Tree SHAP was employed to interpret the prediction of accrual percentage and analyze the importance of individual features with respect to successful RCT recruitment. SHAP is a unified framework to interpret model predictions [33]. It calculates the contribution of each feature to the output, which is defined as the SHAP value equivalent to the Shapley value in game theory. The mean absolute SHAP value of each feature determines the order of importance. In addition to the measure of feature importance, it also identifies whether the impact of a feature on the output is positive or negative. TreeExplainer’s Tree SHAP algorithm was proposed later to estimate the SHAP values specifically for tree-based models [16].

We set specific paired thresholds to discern between the most and least successful recruitment subgroups within the RCTs. The categories were established such that the most successful recruitment group comprised those RCTs with an accrual rate of either ≥ 90%, ≥ 80%, or ≥ 70%, while the least successful recruitment groups were defined by RCTs exhibiting an accrual rate of ≤ 10%, ≤ 20%, or ≤ 30%, respectively, matching each higher threshold with its corresponding lower one. The identified important features were compared between these two subgroups, and their descriptive statistics were also calculated. Continuous variables were evaluated using Mann–Whitney *U* test (two-sided) with Bonferroni correction, and the binary variables were evaluated using Fisher’s Exact test (two-sided) with Bonferroni correction and a cut-off of corrected *p*-value < 0.05 to determine statistical significance.

## Results

### Descriptive Statistics

**Figure 2.**
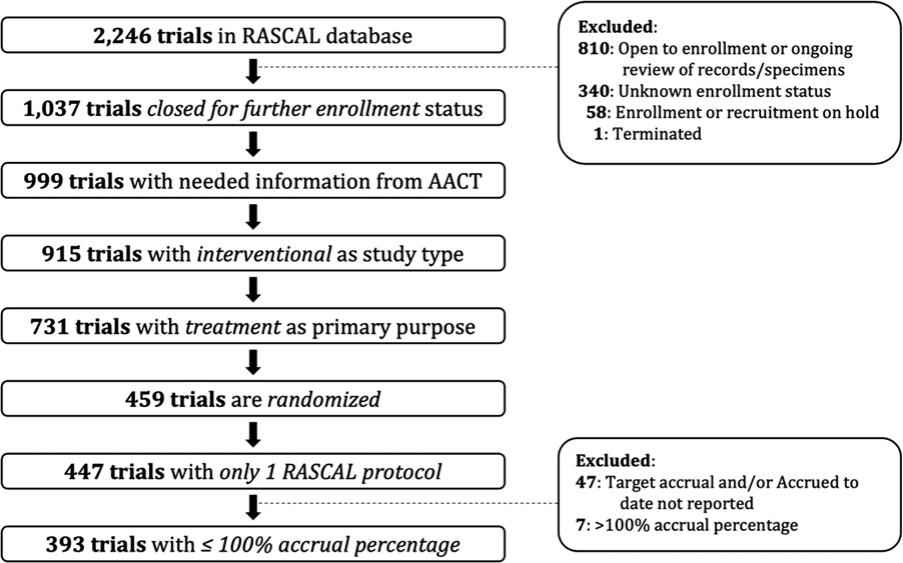
Randomized clinical trials (RCT) selection in research compliance and administration system (RASCAL) and clinicalTrials.gov registries. Each box illustrates the number of RCTs after applying the inclusion and exclusion criteria. AACT = aggregate analysis of clinicaltrials.gov.

Among the 2,246 RCTs in the RASCAL dataset, 1,037 (46%) were closed for further enrollment (the terminated study was excluded). A total of 393 RCTs were included in the analysis (Fig. 2).

The average accrual percentage of the included RCTs is 46.7% (SD: 32.0%). Majority of the RCTs are Phase 3 (*n* = 206; 52.4%), multicenter (96.4%), and industry-funded (67.2%). Most RCTs involve non-English speaking participants (55%), drug or biologic agents (87.5%), collection of biologic specimens (91.9%), and imaging or radiation (61.3%). The most frequently reported recruitment methods are person-to-person (92.9%) and website advertisement (53.9%). Detailed descriptive statistics of the included RCT features are provided in Table 1. The correlations between all analyzed variables are outlined in the accompanying Supplementary Material 2. Key observations include a significant negative correlation of −0.542 between industry-funded studies and protocol duration, suggesting industry trials tend to be shorter. The use of website for recruitment demonstrated high positive correlations with cancer research (coefficient: 0.534) and studies with the target domain of neoplasms (coefficient: 0.493). Cancer research also showed positive associations with studies receiving internal funding (coefficient: 0.436), and the number of sites (coefficient: 0.423), but negatively correlated with studies involving participant compensation (coefficient: −0.723).

**Table 1.** Features of the included RCTs

Features	*n* = 393	Features	*n* = 393
**Protocol duration**, years (median (Q1, Q3) [range])	5 (3, 6) [1–20]	**Number of sites** (median (Q1, Q3) [range])	58 (22, 133) [1–1459]
			
**Target accrual** (median (Q1, Q3) [range])	10 (7, 25) [1–2600]	**Target enrollment** (median (Q1, Q3) [range])	15 (10, 40) [1–6000]
			
**Accrual to date** (median (Q1, Q3) [range])	4 (2, 10) [0–2013]	**Enrolled to date** (median (Q1, Q3) [range])	6 (3, 15) [0–5612]
			
**Accrual percentage** (mean (SD))	46.7 (32.0)	**Number of modifications** (median (Q1, Q3) [range])	1 (0, 2) [0–8]
			
**Study Phase^#^** (*n* (%))		Funding type* (*n* (%))	
Phase 1	19 (4.8)	Federal/State/Local Government	53 (13.5)
Phase 2	138 (35.1)	Industry	264 (67.2)
Phase 3	206 (52.4)	Foundation/Private	8 (2)
Phase 4	13 (3.3)	Internal	50 (12.7)
Not applicable	45 (11.5)	Unknown	23 (5.9)
			
**Multicenter Research** (*n* (%))		Procedures Included in Study* (*n* (%))	
Yes	379 (96.4)	Recording Subjects	42 (10.7)
No	14 (3.6)	Behavioral Intervention	8 (2)
		Biologic Specimens	361 (91.9)
Resource Utilization* (*n* (%))		Cancer Research	139 (35.4)
Clinical Research Resource	107 (27.2)	Drug or Biologic Agent	344 (87.5)
CCPH	1 (0.3)	Genetics Research	173 (44)
None	190 (48.3)	Imaging or Radiation	241 (61.3)
		Medical Device	59 (15)
Involvement & Targeted Populations* (*n* (%))		Surgical Procedures	20 (5.1)
Involves Subject Screening	382 (97.2)		
Involves Sub-Studies	61 (15.5)	Qualitative and Evaluation Methods* (*n* (%))	
Involves Compensation	206 (52.4)	Survey, Interview, Questionnaires¤	277 (70.5)
Minors/Children	51 (13)	Systematic Observation	2 (0.5)
Pregnant Women	8 (2)	Cognitive Test	51 (13)
Lacking Capacity for Consent	52 (13.2)	Education Test	1 (0.3)
CU/NYPH Employees	8 (2)	Noninvasive Measure¤	240 (61.1)
Economically Disadvantaged	36 (9.2)	Taste Test	5 (1.3)
Educationally Disadvantaged	25 (6.4)		
Non-English Speaking	216 (55)	Recruitment Methods Used* (*n* (%))	
Other Vulnerable Population‡	12 (3.1)	Recruitment methods not involved	26 (6.6)
No Vulnerable Population†	140 (35.6)	Person-to-Person	365 (92.9)
		Direct Telephone Calls	32 (8.1)
Written Consent Obtained*(*n* (%))		Radio Advertisements	6 (1.5)
Consent Obtained	388 (98.7)	Newspaper Advertisements	6 (1.5)
Written Consent Waived	19 (4.8)	Direct Mail Invitation	11 (2.8)
Consent Waived per 45CFR46116	3 (0.8)	Website	212 (53.9)
Consent Waived per 21CFR5024	2 (0.5)	Email Invitation	23 (5.9)
Consent Exempt	3 (0.8)	Television Advertisements	5 (1.3)
		Newsletter Advertisements	6 (1.5)
**Written Consent Language** (*n* (%))		Posting on ResearchMatch.org	15 (3.8)
Non-English language expected	234 (59.5)		
Non-English language not expected	158 (40.2)		
Consent Language Unknown	1 (0.3)		

CCPH = columbia community partnership for health; CU = columbia university; NYPH = New York presbyterian hospital; Q1 = first quartile; Q3 = third quartile; SD = standard deviation.

* One RCT may have multiple answers.

# Studies may have multiple phases (e.g., Phase 1/2).

‡ Other unspecified vulnerable population other than Minors/Children, Pregnant Women, Lacking Capacity for Consent, CU/NYPH Employees, Economically Disadvantaged, Educationally Disadvantaged, and Non-English Speaking individuals.

† Studies where the expected enrollment does not specifically include, or aim to recruit from, any recognized vulnerable groups. It does not necessarily imply that these groups are excluded from participation by the eligibility criteria, but rather that they are not the targeted or anticipated demographic for recruitment.

¤ Distinctions between the different types of data collection methods used. Noninvasive measures include the gathering of physiological parameters without the use of invasive procedures, such as monitoring heart rate, measuring blood pressure, or checking temperature. Conversely, ’survey, interview, and questionnaires’ referred to tools utilized to acquire information regarding the participants’ feelings, thoughts, behaviors, or experiences through self-reporting methods. While both categories could be considered ‘noninvasive’ in the broad sense, these were separated due to the distinct types of data each method collects.

The included RCTs represented 43 clinical domains (Table 2), with pathological conditions signs and symptoms as the most commonly targeted domain (36.6%), followed by neoplasms (36.1%) and nervous system diseases (23.2%).

**Table 2.** Target clinical domain for the included rcts according to medical subject headings (MeSH) category extracted from AACT (*n* = 393)

Target Domain Category [MeSH Category]	Count	%
C23: Pathological Conditions, Signs and Symptoms	144	36.6
C04: Neoplasms	142	36.1
C10: Nervous System Diseases	91	23.2
C14: Cardiovascular Diseases	67	17.0
C20: Immune System Diseases	61	15.5
C06: Digestive System Diseases	58	14.8
C17: Skin and Connective Tissue Diseases	57	14.5
C12: Male Urogenital Diseases	39	9.9
C13: Female Urogenital Diseases and Pregnancy Complications	39	9.9
C08: Respiratory Tract Diseases	38	9.7
C15: Hemic and Lymphatic Diseases	37	9.4
C16: Congenital, Hereditary, and Neonatal Diseases and Abnormalities	35	8.9
C18: Nutritional and Metabolic Diseases	35	8.9
C19: Endocrine System Diseases	26	6.6
F03: Mental Disorders	25	6.4
C01: Bacterial Infections and Mycoses	14	3.6
C02: Virus Diseases	10	2.5
C05: Musculoskeletal Diseases	10	2.5
D27: Chemical Actions and Uses	8	2.0
C25: Chemically Induced Disorders	6	1.5
F02: Psychological Phenomena	6	1.5
G04: Cell Physiological Phenomena	6	1.5
G11: Musculoskeletal and Neural Physiological Phenomena	6	1.5
C07: Stomatognathic Diseases	5	1.3
C11: Eye Diseases	5	1.3
C26: Wounds and Injuries	4	1.0
G07: Physiological Phenomena	4	1.0
D02: Organic Chemicals	3	0.8
D04: Polycyclic Compounds	3	0.8
D12: Amino Acids, Peptides, and Proteins	3	0.8
E01: Diagnosis	3	0.8
E05: Investigative Techniques	3	0.8
F01: Behavior and Behavior Mechanisms	3	0.8
D10: Lipids (Amino Acids, Peptides, and Proteins)	2	0.5
B04: Viruses	1	0.3
C09: Otorhinolaryngologic Diseases	1	0.3
D01: Inorganic Chemicals	1	0.3
D06: Hormones, Hormone Substitutes, and Hormone Antagonists	1	0.3
D09: Carbohydrates (Lipids)	1	0.3
D23: Biological Factors	1	0.3
E04: Surgical Procedures, Operative	1	0.3
J02: Food and Beverages	1	0.3
N06: Environment and Public Health	1	0.3

MeSH = medical subject headings.

A single RCT may have multiple target domains.

### Model Performance

Supplementary Table S2 lists the optimal parameter setting for each model. The performances of these eight regression models for accrual percentage prediction under the optimal parameter setting are shown in Table 3. Among them, the CatBoost regressor achieved the smallest mean validation RMSE (20.31, SD: 2.53), and the difference between the mean train and validation RMSE is 5.75 (accrual percentage is within the [0,100]), signifying the model was not overfitted. Therefore, the CatBoost regressor was selected and trained on the whole dataset for feature importance analysis.

**Table 3.** Performances of the eight regression models for accrual percentage prediction

Regression model	Mean validation RMSE (SD)	Mean train RMSE (SD)
Ridge	28.19 (2.75)	23.89 (0.29)
Lasso	27.83 (2.43)	26.08 (0.29)
Decision Tree	25.67 (3.17)	20.96 (0.94)
Random Forest	21.54 (2.67)	16.21 (0.44)
AdaBoost	21.11 (2.57)	14.98 (0.39)
XGBoost	20.64 (2.69)	15.13 (0.28)
LightGBM	20.53 (2.72)	15.03 (0.31)
CatBoost	20.31 (2.53)	14.56 (0.27)

AdaBoost = adaptive boosting; CatBoost = categorical boosting; Lasso = least absolute shrinkage and selection operator; LightGBM = light gradient boosted machine; RMSE = root mean square error; SD = standard deviation; XGBoost = eXtreme gradient boosting.

### Feature Importance Analysis

**Figure 3.**
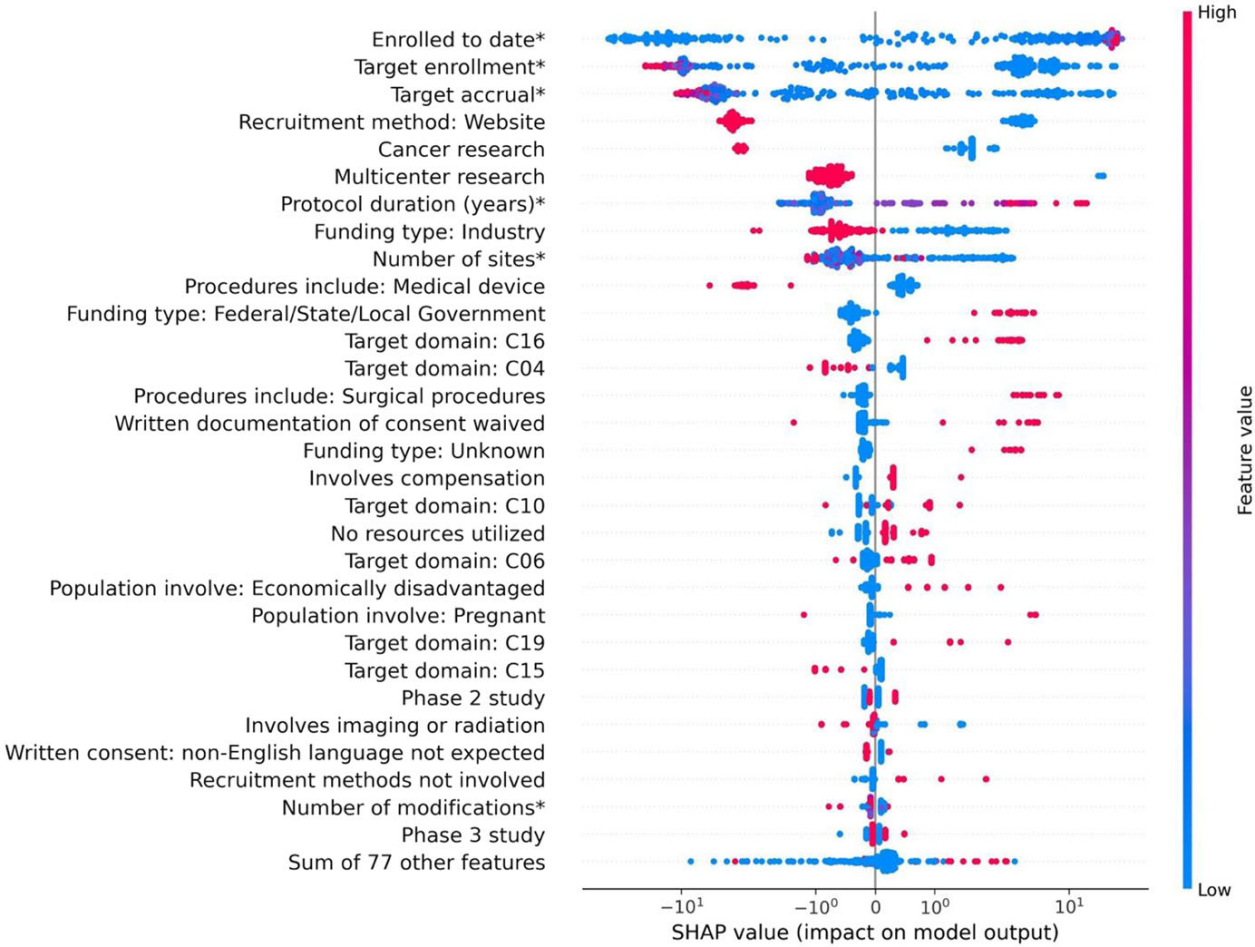
Tree SHapley additive exPlanations (SHAP) summary plot with the Top 30 Most important features associated with RCT recruitment success. The SHAP values have been log scaled. *Features are continuous variables, whereas the others are binary variables. C16: congenital, hereditary, and neonatal diseases and abnormalities. CO4 = neoplasms; C10 = nervous system diseases; C06 = digestive system diseases; C19 = endocrine system diseases; C15 = hemic and lymphatic diseases.

The top 30 most important features that are associated with RCT recruitment based on the CatBoost model are presented in Fig. 3. The top 48 most important features with mean absolute SHAP value > 0.01 are displayed in Supplementary Figure S1. We also provided the important features calculated based on the LightGBM and XGBoost models in Supplementary Figures S2 and S3. The horizontal position of a dot represents the SHAP value of a feature for an RCT. A larger positive (or negative) SHAP value indicates a higher positive (or negative) impact of the feature on the accrual percentage prediction. The color of a dot indicates the feature value. For continuous variables, the redder the dot is, the larger the value is; for binary variables, red indicates the presence of the feature in the RCT.

For the continuous feature “Protocol duration (years),” the higher the value of the feature, the larger the SHAP value (i.e., redder dot) in the positive direction, which indicates a larger positive impact on the accrual percentage. In other words, the RCTs with longer protocol duration in years are more likely to have a high accrual percentage. For the binary feature “Funding type: Federal/State/Local Government,” the SHAP values for RCTs that were funded by the government (red dots) are positive. This indicates that RCTs funded by the government are more likely to have a higher accrual percentage. In contrast, industry-funded RCTs tend to have a lower accrual percentage.

Further, findings show that RCTs with lower target accrual or lower target enrollment are associated with successful recruitment, which is understandable since it is easier to achieve a target with a smaller number of participants. We also found multicenter research tends to have a low accrual percentage. While RCTs involving medical devices were less likely to achieve recruitment success, participant compensation was positively associated with recruitment success. The longer the RCT is active (i.e., the number of protocol years), the more likely it is to accrue participants successfully. Additionally, RCTs not using websites for recruitment are more likely to have a higher accrual percentage. Also, the RCTs involving economically disadvantaged participants are more likely to have a higher accrual percentage. On the other hand, cancer research RCTs and RCTS with target domain C04 (Neoplasms) tend to have a low accrual percentage. RCTS targeting congenital, hereditary, and neonatal diseases and abnormalities (C16) and endocrine system diseases (C19) appears to have a higher percentage accrual.

When comparing the top three prediction models (i.e., CatBoost, LightGBM, and XGBoost; see Fig. 3, Supplementary Figures S2 and S3), all top 30 most important features identified by the CatBoost model are agreed by the XGBoost, and 24 of them are agreed by LightGBM. The features “Multicenter research,” “Population involve: Pregnant,” “Involves imaging or radiation,” “Target domain: C15 (Hemic and Lymphatic Diseases),” “Recruitment method not involved,” and “Written Documentation of consent waived” were deemed important in CatBoost, but not in LightGBM with a mean SHAP value < 0.01.

Finally, in the separate analysis incorporating the seven previously excluded studies, we observed some differences in feature importance (Supplementary Figure S4). Notably, the features “Involves Compensation,” “ Target domain: C10,” “No Resources Utilized,” “ Target domain: C15,” and “Written consent: non-English language not expected” were not identified as important.

### Subgroup Analysis of Most and Least Successful Recruitment

Table 4 summarizes the significant differences (corrected *p*-value < .05) found in multiple features between the worst and best recruitment groups among different successful recruitment cutoffs. Supplementary Tables S3, S4, and S5 provide the details of the comparison.

**Table 4.** Features with a significant difference (Corrected *P*-value < .05) between the best and worst recruitment group under different cutoffs (i.e., ≥ 90% v. ≤ 10%, ≥ 80% vs ≤ 20%, and ≥ 70% vs ≤ 30%) among the top 30 most important features

Cutoff	Feature	Worst recruitment group count, % / Median, (Q1, Q3)	Best recruitment group count, % / Median, (Q1, Q3)	Corrected p-value
≥90% (*n* = 64) vs ≤10% (*n* = 60)	Enrolled to date*	1.0, (0.75, 3.5)	11.0, (4.0, 40.25)	1.53E-23
	Target enrollment*	15.0, (10.0, 40.0)	15.0, (7.75, 60.0)	9.84E-42
	Target accrual*	10.0, (9.5, 25.0)	10.0, (4.0, 33.5)	3.99E-41
	Recruitment method: Website	44, 0.73	15, 0.23	1.08E-06
	Cancer research	32, 0.53	6, 0.09	2.36E-06
	Multicenter research	60, 1.0	54, 0.84	4.17E-02
	Protocol duration (years)*	4.0, (2.0, 5.0)	5.5, (3.0, 9.0)	2.98E-40
	Number of sites*	74.5, (39.75, 138.25)	36.5, (2.75, 88.75)	2.10E-38
	Target domain: C04	32, 0.53	9, 0.14	1.05E-04
	No resources utilized	22, 0.37	43, 0.67	3.35E-02
≥80% (*n* = 87) vs ≤20% (*n* = 110)	Enrolled to date*	2.0, (1.0, 5.0)	11.0, (5.0, 43.5)	4.10E-46
	Target enrollment*	15.0, (10.0, 40.0)	15.0, (8.0, 60.0)	1.55E-66
	Target accrual*	10.0, (8.0, 25.0)	10.0, (5.0, 35.0)	5.22E-66
	Recruitment method: Website	75, 0.68	25, 0.29	1.14E-06
	Cancer research	56, 0.51	8, 0.09	4.11E-09
	Multicenter research	110, 1.0	74, 0.85	4.26E-04
	Protocol duration (years)*	4.0, (2.25, 5.75)	5.0, (3.0, 8.0)	3.30E-64
	Number of sites*	77.5, (39.25, 154.75)	36.0, (7.0, 90.0)	5.14E-62
	Target domain: C16	3, 0.03	16, 0.18	1.07E-02
	Target domain: C04	53, 0.48	11, 0.13	3.25E-06
	Involves compensation	40, 0.36	62, 0.71	5.12E-05
	No resources utilized	44, 0.4	58, 0.67	9.24E-03
	Involves imaging or radiation	78, 0.71	41, 0.47	2.35E-02
≥70% (*n* = 108) vs ≤30% (*n* = 155)	Enrolled to date*	3.0, (1.0, 8.0)	13.0, (6.0, 37.0)	1.98E-68
	Target enrollment*	16.0, (10.0, 40.0)	17.0, (10.0, 60.0)	5.10E-89
	Target accrual*	10.0, (8.0, 25.0)	10.0, (6.0, 33.5)	1.60E-88
	Recruitment method: Website	106, 0.68	34, 0.31	1.48E-07
	Cancer research	78, 0.5	13, 0.12	1.30E-09
	Multicenter research	155, 1.0	94, 0.87	6.86E-05
	Protocol duration (years)*	4.0, (3.0, 6.0)	5.0, (3.0, 7.0)	2.40E-86
	Number of sites*	81.0, (39.5, 156.0)	31.0, (9.5, 88.25)	5.48E-84
	Funding type: Federal/State/Local Government	13, 0.08	29, 0.27	3.58E-03
	Target domain: C16	6, 0.04	18, 0.17	2.20E-02
	Target domain: C04	76, 0.49	16, 0.15	1.50E-07
	Involves compensation	59, 0.38	75, 0.69	2.45E-05
	No resources utilized	62, 0.4	66, 0.61	3.21E-02
	Involves imaging or radiation	111, 0.72	54, 0.5	1.36E-02
	Number of modifications*	1.0, (0.0, 2.0)	0.0, (0.0, 1.0)	3.22E-04

CO4 = neoplasms; C16 = congenital, hereditary, and neonatal diseases and abnormalities.

Mann–Whitney U and Fisher’s Exact tests with Bonferroni correction were used for continuous and binary variables, respectively. The descriptive statistics of each feature for these two subgroups are also listed.

* Features are continuous variables where the median, the first quantile (Q1), and the third quantile (Q3) were calculated, whereas the others are binary variables where the count and the percentage were calculated.

At the cutoff of ≥ 90% (*n* = 64) vs ≤ 10% (*n* = 60), the “Enrolled to date” and “Target enrollment” were notably different with median values of 1.0 and 15.0 for the worst recruitment group and 11.0 and 15.0 for the best recruitment group, respectively. The feature “Cancer research” has a negative association with the accrual percentage (also see Fig. 3), as demonstrated by having more RCTs in the worst recruitment group compared to the best recruitment group (53% vs 9%). The use of a website as a recruitment method was more commonly used in the worst recruitment group. Multicenter research was also more prevalent in the worst recruitment group. Studies that did not utilize available resources were significantly more common in the best recruitment group. Further, the target accrual, protocol duration in years, number of sites, and studies with target domain neoplasms all differed between the two groups.

When the cutoff was adjusted to ≥ 80% (*n* = 87) vs ≤ 20% (*n* = 110), additional differences emerged in studies with target domain congenital, hereditary, and neonatal diseases and abnormalities. Studies involving compensation were more prevalent in the best recruitment group while studies involving imaging or radiation were more common in the worst recruitment groups. At the final cut-off of ≥ 70% (*n* = 108) vs ≤ 30% (*n* = 155), new differences were also observed in the prevalence of studies funded by government agencies and the number of modifications.

## Discussion

In this retrospective data analysis using machine learning methods, we examined the factors associated with RCT recruitment success based on the accrual percentage. Overall, the accrual percentage of our sample RCTs confirms the high frequency of participant recruitment challenges [34]. Consistent with the mixed evidence of how the funding type is associated with accrual [35–37], our results demonstrated that successful recruitment varies widely by funding type. In the feature importance analysis, we found that government-funded RCTs are more likely to be successful, while industry-funded studies are less likely to be successful. However, we found government funding to be significant for studies with accrual percentages of ≥ 70% and ≤ 30%. The notable negative correlation (coefficient: −0.542) observed between industry-funded studies and protocol duration corroborates the idea that industry-sponsored trials often adopt a faster pace, possibly due to higher resource availability or stricter time constraints [36,37].

Another key finding in this study is the negative association of the multicenter research feature with the accrual percentage. While this finding does not allow us to definitively gauge the overall success of multicenter RCTs beyond individual institutional accrual, it does imply individual sites recruit easier on single-site RCTs than for multi-site RCTs with the latter imposing more complexities and constraints, despite that multi-site RCTs may scale easily and recruit more participants quickly. Given their manageable sample size and relatively more flexible recruitment strategies that can be customized to the specific locale, single-site RCTs may exhibit higher likelihoods of success [38]. Notably, multicenter research did not demonstrate any substantial correlations with the other variables under investigation in this study.

In examining the target domain of the RCTs, our findings confirm that recruitment for oncology research presents more challenges than other fields, potentially due to high patient competition or stringent eligibility criteria, corroborating previous studies [7,39]. However, the cancer research domain also displayed positive correlations with studies receiving internal funding (coefficient: 0.436) and those involving a larger number of sites (coefficient: 0.423), likely reflecting the high societal and clinical impact of these studies. Curiously, a negative correlation was found between cancer research and participant compensation (coefficient: −0.723), possibly suggesting that potential health benefits or access to novel therapies in cancer research can supersede financial incentives for participants. In a surprising turn, both feature importance and subgroup analyses demonstrated that RCTs targeting congenital, hereditary, and neonatal diseases and abnormalities tend to be successful. Despite inherent recruitment challenges for rare diseases [39,40], factors such as targeted sample size, well-organized patient communities, specialized research institutions, and limited treatment availability – which in turn heightens the value of clinical trials for patients – may have contributed to their success [41].

The procedure involvement required by the RCT can also influence recruitment success, as demonstrated by how the involvement of medical devices negatively influences accrual percentage. Perceived drawbacks of participating in research and perception of therapeutic benefit may have directly affected the likelihood of patient enrollment [12]. Further, we found in both analyses that proper compensation was associated with better recruitment. The observation that adequate participant compensation is associated with improved recruitment corroborates previous studies and underscores the salient role of compensation in motivating potential participants, especially among economically disadvantaged populations [21]. This could also contribute to why RCTs involving economically disadvantaged participants tend to be successful, as compensation can be an essential incentive for encouraging participation, particularly for individuals with financial constraints or other barriers to participation (e.g., commute to study site, missing work) [42]. However, this relationship necessitates ethical vigilance. A paramount concern is the possibility of undue inducement, where the attractiveness of the compensation might lead potential participants to disregard the potential risks associated with the trial or undermine the voluntariness of their participation [43]. Further, the distribution of compensation warrants scrutiny to guard against any unintentional exploitative practices or the inadvertent exclusion of certain demographic groups from trial participation [44]. Hence, while compensation can act as a potent recruitment tool, its deployment should be governed by a conscientious adherence to the principles of respect for persons, beneficence, and justice, as outlined in seminal ethical guidelines such as the Belmont Report [45].

Lastly, we did not find any recruitment method that is positively associated with accrual, implying there is no “one-fitting-all” solution for recruitment so investigators should also analyze the recruitment situation case by case and seek appropriate methods. A flexible infrastructure for recruitment is needed. Though the person-to-person recruitment method is the most commonly used (93%), it did not demonstrate an association with the accrual percentage. However, previous evidence shows that person-to-person recommendations tend to be trusted more than other methods and can influence a potential participant’s decision to participate in an RCT [46]. In line with previous findings [47], the use of websites, direct mail, and television advertisements for recruitment was negatively associated with accrual percentage. A possible explanation could be that the RCTs struggling with recruitment may exploit more recruitment strategies to expand their outreach. Furthermore, research teams may have other strategies (e.g., chart reviews [37], clinician engagement [14]) that are outside the scope of our data.

### Recommendations for Recruitment Improvement

A critical area for increasing recruitment success is focusing overall recruitment strategies based on the population of interest. Previous research efforts have highlighted how passive recruitment methods leveraging novel technologies (e.g., online advertisements, web-based screening tools) can drastically reduce the time and cost associated with clinical trial coordination; however, the effectiveness can depend on the potential participant’s time online and computer literacy levels [48]. Although technology provides a wide array of novel recruitment methods, community engagement may be more beneficial depending on the population. For example, personal and community-focused strategies have been successful in racial and ethnic minority populations recruitment [49]. Recruitment methods that demonstrated a negative association (i.e., website, radio, direct mail, and television; see Supplementary Figure S1) with recruitment success should be employed with the understanding that these methods alone may not be sufficient.

Furthermore, planning and implementing a flexible recruitment infrastructure and a comprehensive approach to recruitment is necessary for studies with challenges in accrual (e.g., oncology, medical device involvement, imaging, and radiation involvement). Hence, it is not just about casting the net wide; it’s about casting it smartly, which involves several key aspects. One, we need to ensure we have the appropriate funds allocated to our recruitment efforts. Two, we must invest in the proper training for our clinical research staff so they are equipped to handle nuanced recruitment strategies. And three, patient education is crucial. We need to make sure potential participants understand the trial, its benefits, and its risks.

Lastly, and quite importantly, our research underscores the significant benefit of fairly compensating participants. While our results indicate that patient compensation is associated with higher accrual, we cannot make a definitive recommendation for increasing patient compensation as a strategy to enhance recruitment. Rather, we suggest that trial designers consider our findings as one piece of a complex puzzle when planning their recruitment strategies. Participant compensation not only aids recruitment but also helps reduce the burden on those who participate in these trials, particularly for individuals who may have to travel long distances or miss work to participate in the RCT. Compensation can help ensure that our trials are as inclusive and equitable as possible, by enabling a more diverse range of participants.

By optimizing recruitment strategies, trials can be made more cost-effective, and most importantly, diverse. Therefore, greater emphasis on a thoughtful and successful implementation of these novel recruitment strategies could serve as an essential step for future improvement in recruitment practices.

### Strengths, Limitations, and Next Steps

This study has several strengths and limitations. To our knowledge, this is the first study to use a data-driven method to systematically identify factors associated with recruitment successes across disease types, trial designs, recruitment methods, funding types, and patient population (e.g., non-English speaking, economically disadvantaged). However, due to the nature of the retrospective analysis, we were unable to establish causality between the collected features and successful patient recruitment. Further, though we used multiple data sources, the RCTs analyzed are from a single institution; hence, future studies are warranted to test the generalizability of the findings to other institutions. In addition, we were unable to include features that have previously shown substantial influence on clinical trial enrollment, such as the number of competing trials and eligibility criteria complexity due to the incompleteness of the information in our dataset [50]. Additionally, since studies may not report all recruitment methods and characteristics, our findings could be affected by potential underreporting or incomplete data; this should be considered when interpreting the results. Besides, we made an effort to tune the parameters of models to improve their performances, but there may be additional configurations that we did not explore that could lead to further improvement. Future work in this field should include more longitudinal data collection, improved automated natural language processing, and a greater expansion of trial information for modeling to address these stated limitations and further enhance our understanding of patient recruitment. Finally, assessing the impact of our suggested actions is crucial for validating their effectiveness in enhancing participant recruitment, allowing for a stronger appraisal of our recommendations’ potential benefits.

## Conclusion

With continuing challenges in accruing sufficient participants for RCTs, it is imperative to investigate the factors influencing recruitment success to develop more effective solutions. This multi-source retrospective study demonstrated key features that are positively (e.g., government funding, compensation, and target domains on congenital, hereditary, and neonatal diseases and abnormalities) and negatively (e.g., cancer research, recruitment methods) associated with participant recruitment into RCTs. Further, multicenter RCTs tended to have poor accrual percentages in a single institution. Finally, actionable steps are provided to allow clinical researchers and research centers to improve participant recruitment in the future. Though further exploration of the causative relationships between the features and successful recruitment, the scope of this analysis is unprecedented and provides greater generalizability to its findings than previously reported. It also leverages machine learning approaches for assessing various RCT features, strengthening future research efforts in this space.

## Supporting information

Idnay et al. supplementary material 1 Idnay et al. supplementary material

Idnay et al. supplementary material 2 Idnay et al. supplementary material

## Supplementary material

The supplementary material for this article can be found at https://doi.org/10.1017/cts.2023.623.
