# Supplementary material for: Uncovering key clinical trial features influencing recruitment
Source: J Clin Transl Sci. 2023 Sep 4;7(1):e199. doi: 10.1017/cts.2023.623 (PMC10565197; doi:10.1017/cts.2023.623)
Supplement: Idnay et al. supplementary material 1 — Idnay et al. supplementary material [file S2059866123006234sup001.docx]

**Uncovering Key Clinical Trial Features Impacting Recruitment**

**Supplementary Materials**

**Table S1.** Description of the features and the rationale for selection based on domain expertise and existing evidence. Features in bold were included in the model.

| Features | | | Description | Rationale | |
| --- | --- | --- | --- | --- | --- |
| 1 | OID | Unique identification number | | Not relevant |  |
| 2 | Protocol number | Unique protocol number | | Not relevant |  |
| 3 | **Protocol year** | Nth year of the protocol since entered into RASCAL | | Provides information on how long it has been since the protocol was submitted to the IRB. |  |
| 4 | **Number of modifications** | Nth number of protocol modification | | Provides information on how often the protocol was modified. |  |
| 5 | User ID | Unique user identification number of the person who prepared the protocol submission | | Not relevant |  |
| 6 | First name | First name of the person who prepared the protocol submission | | Not relevant |  |
| 7 | Last name | Last name of the person who prepared the protocol submission | | Not relevant |  |
| 8 | Originating department | Department affiliation of the project | | Departments may have different strategies or infrastructure for recruitment. |  |
| 9 | Title | Title of the study | | Not relevant |  |
| 10 | **Multicenter research** | Yes/No: Is this research part of a multicenter study? | | A greater number of sites allows for exposure to more potential participants^1^ |  |
| 11 | Resource utilized: CPDM | Yes/No: University resource utilization of Cancer Center Clinical Protocol Data Management Compliance Core (CPDM) | | Excluded because this resource is only available to cancer clinical research. |  |
| 12 | **Resource utilized: CRR** | Yes/No: University resource utilization of CTSA-Irving Institute Clinical Research Resource (CRR) | | Organizational support may impact recruitment^2^ |  |
| 13 | **Resource utilized: CCPH** | Yes/No: University resource utilization of CTSA- Irving Institute Columbia Community Partnership for Health (CCPH) | |  |  |
| 14 | **No resources utilized** | Yes/No: No university resources were utilized | |  |  |
| 15 | Total staff count | Total number of personnel (e.g., principal investigator, investigator, research coordinator, other engaged personnel) | | The numbers were not aggregated based on the personnel role; hence will result in an incorrect inference (i.e., unclear how many personnel are involved in recruitment). Further, the literature focuses on the research staff’s cultural representation and language concordance, not the number. ^3,4^ |  |
| 16 | Clinical trial registration number | National Clinical Trial (NCT) Identification number | | Not relevant |  |
| 17 | Procedures include: Analysis of existing data | Yes/No: Study procedures involved analysis of existing data and/or prospective record review | | Not relevant |  |
| 18 | **Procedures include: Recordingsubjects** | Yes/No: Study procedures involved audio and/or video recording of research subjects | | Study design may impact recruitment. ^3,5,6^ |  |
| 19 | **Procedures include: Behavioral intervention** | Yes/No: Involved behavioral Intervention | |  |  |
| 20 | **Procedures include: Biological specimens collection** | Yes/No: Involved collection or use of biological specimens | |  |  |
| 21 | **Procedures include: Cancer research** | Yes/No: Involved cancer-related research | |  |  |
| 22 | **Procedures include: Drug or biologic evaluation** | Yes/No: Involved drugs or biologics | |  |  |
| 23 | **Procedures include: Genetic research** | Yes/No: Involved genetic research | |  |  |
| 24 | **Procedures include: Human embryo** | Yes/No: Involved human embryos or human embryonic stem cells | |  |  |
| 25 | **Procedures include: Imaging or radiation** | Yes/No: Involved imaging procedures or radiation | |  |  |
| 26 | **Procedures include: Medical device** | Yes/No: Involved medical devices | |  |  |
| 27 | **Procedures include: Surgical procedures** | Yes/No: Involved surgical procedures that would not otherwise be conducted or are beyond standard of care | |  |  |
| 28 | **Procedures include: Survey, interview, questionnaires** | Yes/No: Involved survey, interview, or questionnaire | |  |  |
| 29 | **Procedures include: Systematic observation** | Yes/No: Involved systematic observation of public or group behavior | |  |  |
| 30 | **Procedures include: Cognitive testing** | Yes/No: Involved cognitive testing | |  |  |
| 31 | **Procedures include: Educational testing** | Yes/No Involved educational testing | |  |  |
| 32 | **Procedures include: Noninvasive measurements** | Yes/No: Involved noninvasive physical measurements | |  |  |
| 33 | **Procedures include: Taste testing** | Yes/No: Involved taste testing | |  |  |
| 34 | Procedures include: Program evaluation | Yes/No: Study procedures involved program evaluation | | Not relevant because our focus is on RCT. |  |
| 35 | Describe recruitment plans | Free text: Describe plans for screening and/or determining the eligibility of prospective subjects | | Requires a different type of analysis that is out of scope for the present study. |  |
| 36 | Recruitment method description | Free text: Describe how participants will be recruited | |  |  |
| 37 | **Recruitment methods not involved** | Yes/No: Recruitment methods not involved | | The impact of the types of recruitment methods may vary^4^ |  |
| 38 | **Recruitment method: Person-to-person** | Yes/No: Involved person to person recruitment | |  |  |
| 39 | **Recruitment method: Radio** | Yes/No: Involved radio advertisement | |  |  |
| 40 | **Recruitment method: Newspapers** | Yes/No: Involved newspaper advertisement | |  |  |
| 41 | **Recruitment method: Direct mail** | Yes/No: Involved direct mail invitation | |  |  |
| 42 | **Recruitment method: Website** | Yes/No: Involved website advertisement | |  |  |
| 43 | **Recruitment method: Email** | Yes/No: Involved email invitation | |  |  |
| 44 | **Recruitment method: Television** | Yes/No: Involved television advertisement | |  |  |
| 45 | **Recruitment method: Telephone** | Yes/No: Involved telephone call invitation | |  |  |
| 46 | **Recruitment method: Newsletter** | Yes/No: Involved newsletter advertisement | |  |  |
| 47 | **Recruitment method: ResearchMatch posting** | Yes/No: Involved ResearchMatch.org posting | |  |  |
| 48 | Recruitment method: RecruitMe posting | Yes/No: Involved RecruitMe (recruit.cumc.columbia.edu) posting | | This resource is only available to a single site. |  |
| 49 | **Written consent will be obtained** | Yes/No: Informed consent with written documentation will be obtained from the research participant or appropriate representative. | | The consent process may impact recruitment. ^5^ |  |
| 50 | **Written consent: waived** | Yes/No: Informed consent will be obtained, but a waiver of written documentation of consent (i.e., agreement to participate in the research without a signature on a consent document) is requested. | |  |  |
| 51 | **Written consent: Waive per 45cfr46116 requirement** | Yes/No: A waiver of some or all elements of informed consent (45 CFR 46.116) is requested. | |  |  |
| 52 | **Written consent: Exception 21cfr5024 emergency** | Yes/No: Planned Emergency Research with an exception from informed consent as per 21 CFR 50.24. | |  |  |
| 53 | **Exempt research** | Yes/No: This is exempt research. | |  |  |
| 54 | **Written consent: non-English language expected** | Yes/No: Informed consent non-English language expected | |  |  |
| 55 | **Written consent: non-English language not expected** | Yes/No: Informed consent non-English language is not expected | |  |  |
| 56 | **Written consent: language unknown** | Yes/No: Informed consent language unknown irrelevant (e.g., record reviews, mass mailing of surveys) | |  |  |
| 57 | **Target enrollment** | Target number of enrollment | | Outcomes of interest |  |
| 58 | **Enrolled to date** | Number of participants enrolled to date | |  |  |
| 59 | **Enrolled since last renewal** | Number of participants enrolled since the last renewal | |  |  |
| 60 | **Anticipated enrolled** | Number anticipated to be enrolled in the next approval period | |  |  |
| 61 | **Target accrual** | Target number of accrual | |  |  |
| 62 | **Accrued to date** | Number of participants accrued to date | |  |  |
| 63 | **Study involves screening** | Yes/No: Does this study involve screening/assessment procedures to determine subject eligibility? | | Common strategy employed by research staff to help with recruitment. ^7,8^ |  |
| 64 | **Study have substudies** | Yes/No: Does this study have one or more components that apply to a subset of the overall study population (e.g., Phase 1/2, sub-studies)? | | Study design may impact recruitment. ^3,5,6^ |  |
| 65 | **Population involve: Minors** | Yes/No: Will children/minors be enrolled | | The target population may impact recruitment. ^3,4,6,9^ |  |
| 66 | **Population involve: Pregnant** | Yes/No: Will pregnant women/fetuses/neonates be targeted for enrollment? | |  |  |
| 67 | **Population involve: Prisoners** | Yes/No: Prisoners targeted for enrollment | |  |  |
| 68 | **Population involve: Lacking capacity to consent** | Yes/No: Involves individuals lacking the capacity to provide consent | |  |  |
| 69 | **Population involve: CU/NYPH Affiliates** | Yes/No: Involves Columbia University or New York Presbyterian Hospital Employees/Residents/Fellows/Interns/Students | |  |  |
| 70 | **Population involve: Economically disadvantaged** | Yes/No: Involves economically disadvantaged | |  |  |
| 71 | **Population involve: Educationally disadvantaged** | Yes/No: Involves educationally disadvantaged | |  |  |
| 72 | **Population involve: Non-English speaking** | Yes/No: Involves non-English speakers | |  |  |
| 73 | **Population involve: Other vulnerable populations** | Yes/No: Involves other vulnerable populations | |  |  |
| 74 | **No vulnerable population targeted** | Yes/No: None of the Populations listed above will be targeted for Enrollment | |  |  |
| 75 | **Involves compensation** | Yes/No: Does this study involve compensation or reimbursement to subjects? | | Remuneration may help recruitment. ^10^ |  |
| 76 | Age distribution: 0-7 | Of the number enrolled, or the number accrued for interventional studies with a screening process, the percent of participants in the age range | | We did not include this because we cannot distinguish if the number reported is enrolled or accrued. |  |
| 77 | Age distribution: 8-17 |  |  |  |  |
| 78 | Age distribution: 18-65 |  |  |  |  |
| 79 | Age distribution: >65 |  |  |  |  |
| 80 | Age distribution: not specified |  |  |  |  |
| 81 | Ethnicity distribution: Hispanic or Latino | Of the number enrolled, or the number accrued for interventional studies with a screening process, the percentage of participants categorized as Ethnic Hispanic or Latino, Ethnic Not Hispanic or Latino, or Ethnic Non-Specific | |  |  |
| 82 | Ethnicity distribution: Not Hispanic or Latino |  |  |  |  |
| 83 | Ethnicity distribution: not specified |  |  |  |  |
| 84 | Gender distribution: Females | Of the number enrolled, or the number accrued for interventional studies with a screening process, the percentage of participants categorized as Gender Females, Males, or Non-Specific | |  |  |
| 85 | Gender distribution: Males |  |  |  |  |
| 86 | Gender distribution: not specified |  |  |  |  |
| 87 | Race distribution: American Ind/Alaskan | Of the number enrolled, or the number accrued for interventional studies with a screening process, the percentage of participants categorized as Race American Indian/Alaskan, Asian, Native Hawaiian or Other, Black or African American, White, More than One Race, or Non-Specific | |  |  |
| 88 | Race distribution: Asian |  |  |  |  |
| 89 | Race distribution: Native Hawaiian or Other |  |  |  |  |
| 90 | Race distribution: Black or African Am |  |  |  |  |
| 91 | Race distribution: White |  |  |  |  |
| 92 | Race distribution: More than One Race |  |  |  |  |
| 93 | Race distribution: not specified |  |  |  |  |
| 94 | **Funding type** | Any external funding or support that is applied for or awarded for this project: Federal/State/Local Government, Industry, Foundation/Private, or Internal funding | | Patient recruitment varies widely by sponsor type. ^11-15^ |  |
| 95 | Funding source | Name of awarding agency | | Not relevant |  |
| 96 | Location type | Location type: Columbia/CUMC, NYP Hospital, or Offsite | | Not relevant. It does not provide information regarding the site to infer the impact on recruitment. |  |
| 97 | Facility name | Facility name | | Not relevant |  |
| 98 | Drug name | Investigational drug name | |  |  |
| 99 | Manufacturer name | Investigational drug manufacturer | |  |  |
| 100 | Manufacturer address | Investigational drug manufacturer’s address | |  |  |
| 101 | Manufacturer contact | Investigational drug’s manufacturer’s contact information | |  |  |
| 102 | Dose | Investigational drug dose | |  |  |
| 103 | **Study phase** | Study Phase. A clinical trial can have multiple phases. | | Study design may impact recruitment. ^3,5,6^ |  |
| 104 | **Number of sites** | The number of study sites. | | A greater number of sites allows for exposure to more potential participants^1^ |  |
| 105 | **Target clinical domain** | The medical subject heading (MeSH) category of the disease domain. | | The target population may impact recruitment. ^3,4,6,9^ |  |

**Data Preprocessing**

The granularity of the funding type classification was adjusted from “Industry,” “Federal/State/Local Government,” “Internal Sponsored Project,” “External Pharmaceutical,” “Foundation/Private,” “External Federal Agency,” “External,” “External Medical Device,” “Gift/Endowment,” and “Internal endowment,” to “Federal/State/Local Government,” “Industry,” “Foundation/Private,” and “Internal funding.”

For all binary variables, such as resource utilization and recruitment method classes of features, we assumed that a missing value indicates the absence of a feature of a clinical trial. One-hot encoding was applied to polytomous variables (categorical variables with more than two possible values), including “Funding type,” “Study phase,” and “Target clinical domain.”

The target clinical domain of a clinical trial was extracted from the relevant MeSH terms displayed on ClinicalTrials.gov. The target clinical domain of an RCT was extracted from the relevant Medical Subject Headings (MeSH) terms displayed on ClinicalTrials.gov. MeSH are standardized keywords from a controlled and hierarchically-organized vocabulary produced by the National Library of Medicine and is publicly available at <http://www.ncbi.nlm.nih.gov/pubmed/advanced>. For those without a relevant MeSH term (e.g., NCT02387970, NCT02175641), we manually mapped the conditions of a trial to MeSH terms and retrieved their categories.

**Table S2.** The optimal parameter setting of each model.

| Regression Model | Optimal Parameter Setting | Description |
| --- | --- | --- |
| Ridge | max_iter = 5000 alpha = 10 | Parameter max_iter was fixed. Parameter alpha was tuned. |
| Lasso | max_iter = 5000 alpha = 0.5 | Parameter max_iter was fixed. Parameter alpha was tuned. |
| Decision Tree | max_depth = 5 min_samples_split = 16 | Parameters max_depth and min_sample_split were tuned. |
| Random Forest | max_depth = 5 min_samples_split = 4 max_features = None | Parameters max_depth, min_sample_split, and max_features were tuned. |
| AdaBoost | loss='square' base_estimator = DecisionTreeRegressor(max_depth=5, min_samples_split=32) learning_rate = 0.1 n_estimators = 100 | Parameters loss, learning_rate, and n_estimators were fixed. Parameter base_estimator were tuned. |
| XGBoost | colsample_bytree = 0.8 eta = 0.1 gamma = 1 max_depth = 6 min_child_weight = 30 n_estimators = 100 reg_alpha = 10 reg_lambda = 10 subsample = 0.8 | Parameters eta and n_estimators were fixed. Parameters colsample_bytree, gamma, max_depth, min_child_weight, reg_alpha, reg_lambda, and subsample were tuned. |
| LightGBM | eval_metric = 'RMSE' learning_rate = 0.1 n_estimators = 100 colsample_bytree = 0.8 max_depth = 4 min_split_gain = 10 num_leaves = 5 reg_alpha = 10 reg_lambda = 10 subsample = 0.8 | Parameters eval_metric, learning_rate, n_estimators were fixed. Parameters colsample_bytree, max_depth, min_split_gain, num_leaves, reg_alpha, reg_lambda, and subsample were tuned. |
| CatBoost | iterations = 100 learning_rate = 0.1 depth = 3 reg_lambda = 1 | Parameters iterations and learning_rate was fixed. Parameter depth and reg_lambda were tuned. |

*Note***.** We used Python packages SciKit-Learn v1.1.3 for Ridge, Lasso, Decision Tree, Random Forest, and AdaBoost, xgboost v1.7.1 for XGBoost, lightgbm v3.3.3 for LightGBM, and catboost v1.1.1 for CatBoost.

**Figure S1.** SHapley Additive exPlanations (SHAP)summary plot with the top 48 most important features (mean absolute SHAP value of a feature > 0.01) based on the CatBoost model for successful trial recruitment.
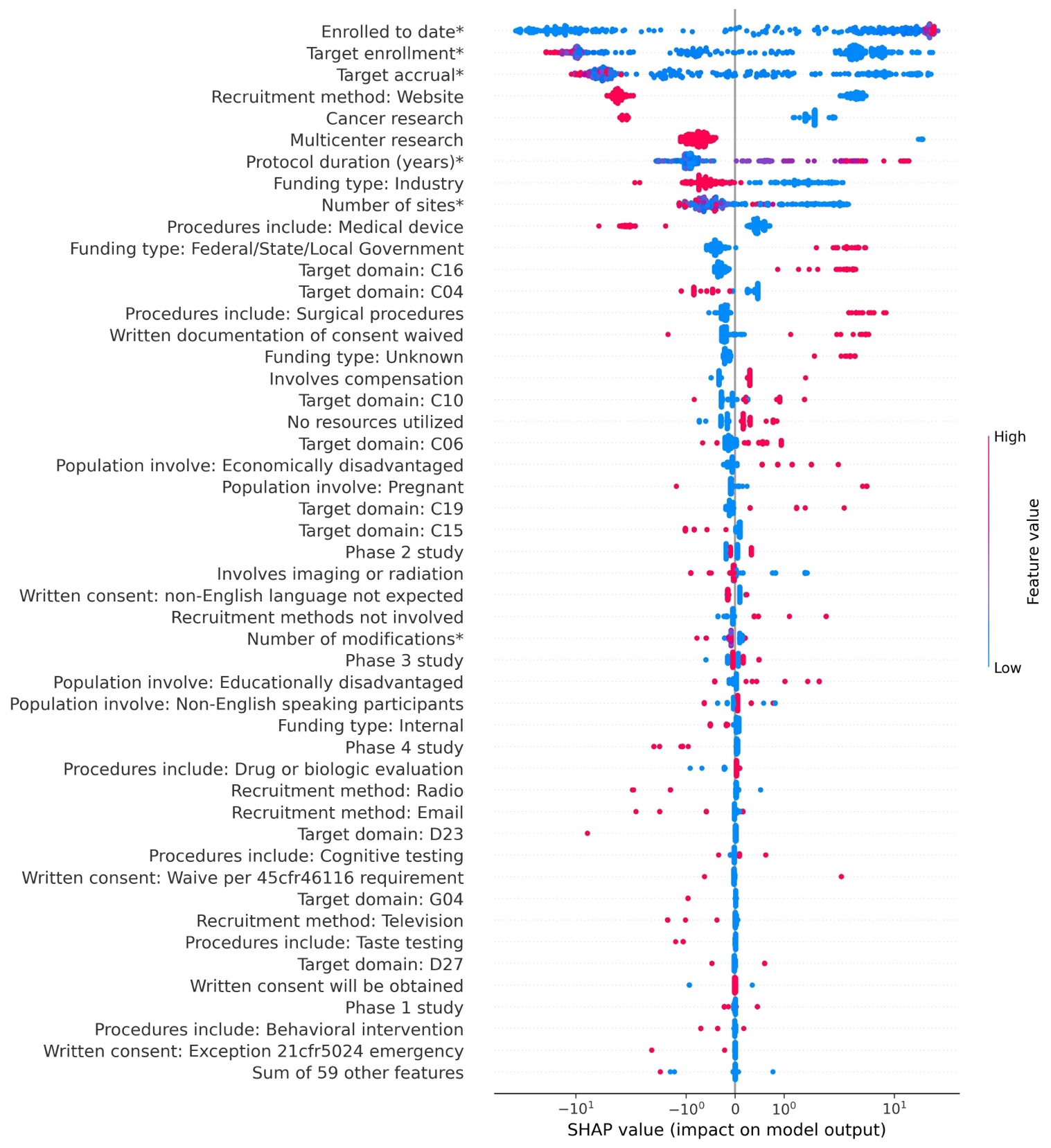


*Note*: * Features are continuous variables, whereas the others are binary variables. The SHAP values have been log scaled. C16: Congenital, hereditary, and neonatal diseases and abnormalities. CO4: Neoplasms. C10: Nervous System Diseases. C06: Digestive System Diseases. C19: Endocrine System Diseases. C15: Hemic and lymphatic diseases. D23: Biological Factors. G04: Cell Physiological Phenomena. D27: Chemical Actions and Uses.

**Figure S2.** SHapley Additive exPlanations (SHAP)summary plot with the top 34 most important features (mean absolute SHAP value of a feature > 0.01) based on the LightGBM model for successful trial recruitment.


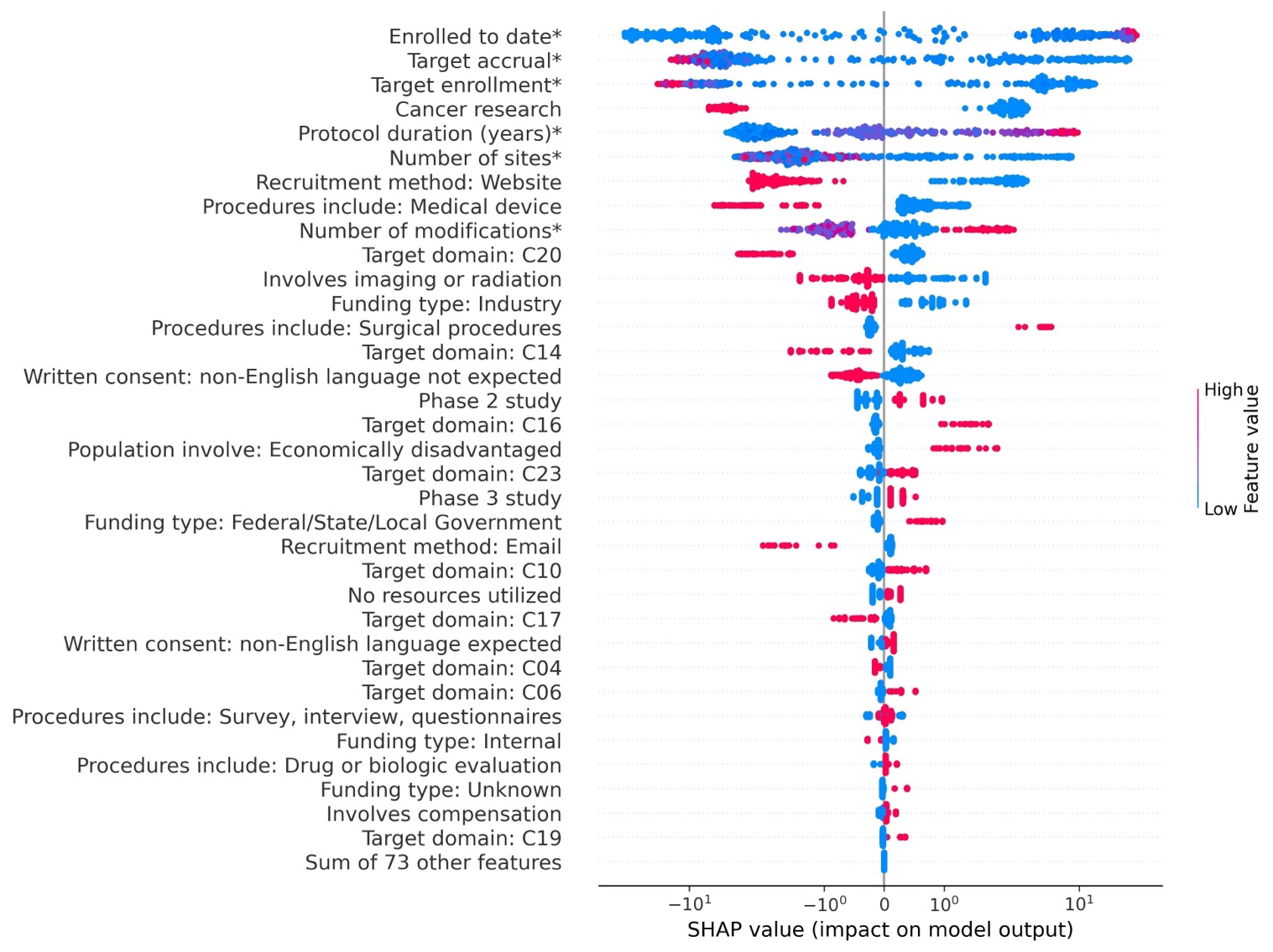


*Note*: * Features are continuous variables, whereas the others are binary variables. The SHAP values have been log scaled. C20: Immune System Diseases. C14: Cardiovascular Diseases. C16: Congenital, hereditary, and neonatal diseases and abnormalities. C23: Pathological Conditions, Signs and Symptoms. C10: Nervous System Diseases. C17: Skin and Connective Tissue Diseases. CO4: Neoplasms. C06: Digestive System Diseases. C19: Endocrine System Diseases.

**Figure S3.** SHapley Additive exPlanations (SHAP)summary plot with the top 80 most important features (mean absolute SHAP value of a feature > 0.01) based on the XGBoost model for successful trial recruitment.


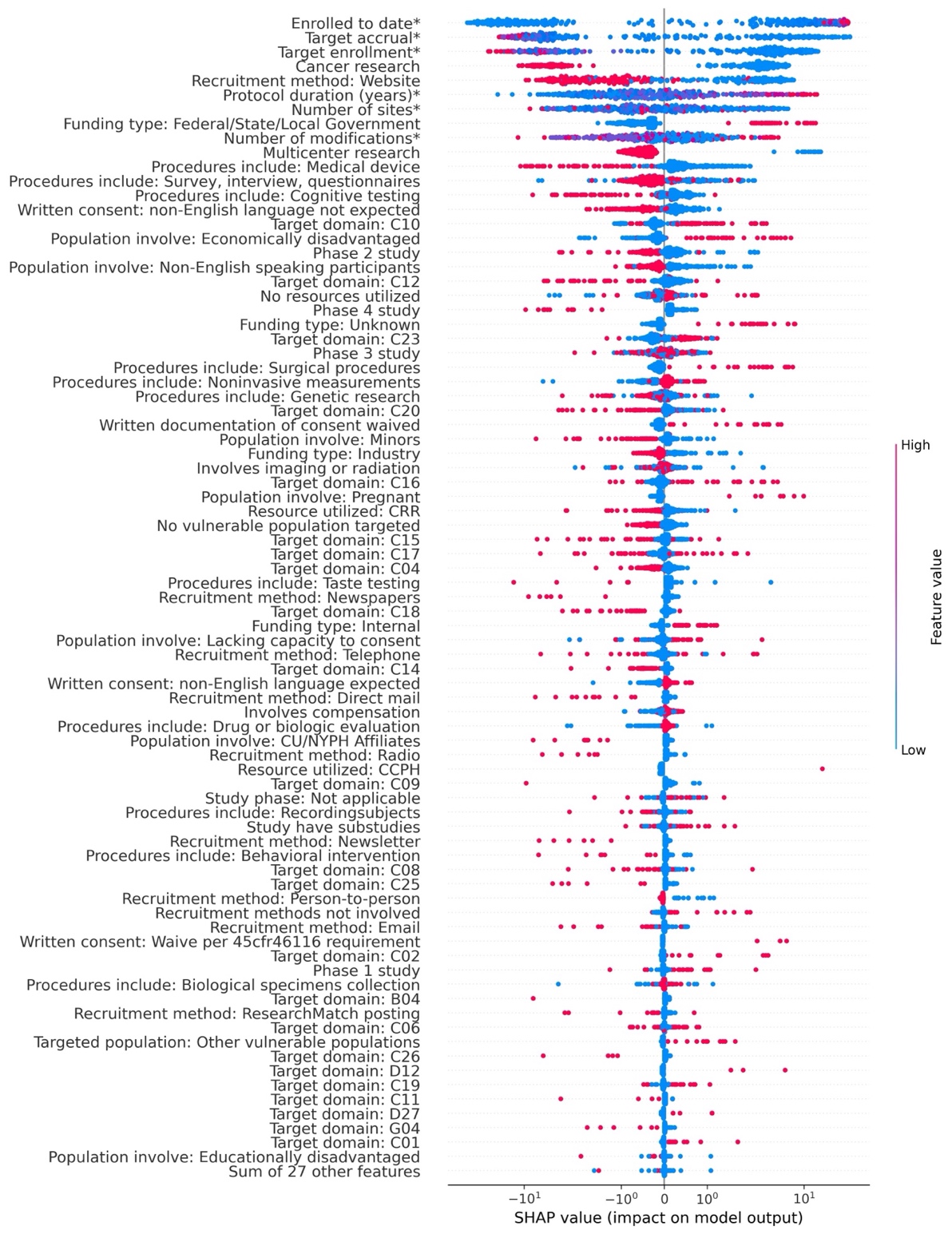


*Note*: * Features are continuous variables, whereas the others are binary variables. The SHAP values have been log scaled. C10: Nervous System Diseases. C12: Male Urogenital Diseases. C23: Pathological Conditions, Signs and Symptoms. C20: Immune System Diseases. C16: Congenital, hereditary, and neonatal diseases and abnormalities. C15: Hemic and Lymphatic Diseases. C17: Skin and Connective Tissue Diseases. C04: Neoplasms. C18: Nutritional and Metabolic Diseases. C14: Cardiovascular Diseases. C09: Otorhinolaryngologic Diseases. C08: Respiratory Tract Diseases. C25: Chemically-Induced Disorders. C02: Virus Diseases. B04: Viruses. C06: Digestive System Diseases. C26: Wounds and Injuries. D12: Amino Acids, Peptides, and Proteins. C19: Endocrine System Diseases. C11: Eye Diseases. D27: Chemical Actions and Uses. G04: Cell Physiological Phenomena. C01: Bacterial Infections and Mycoses.

**Figure S4.** SHapley Additive exPlanations (SHAP)summary plot with the top 40 most important features (mean absolute SHAP value of a feature > 0.01) and 400 trials (including 7 trials with accrual percentage >100%) based on the CatBoost model for successful trial recruitment.


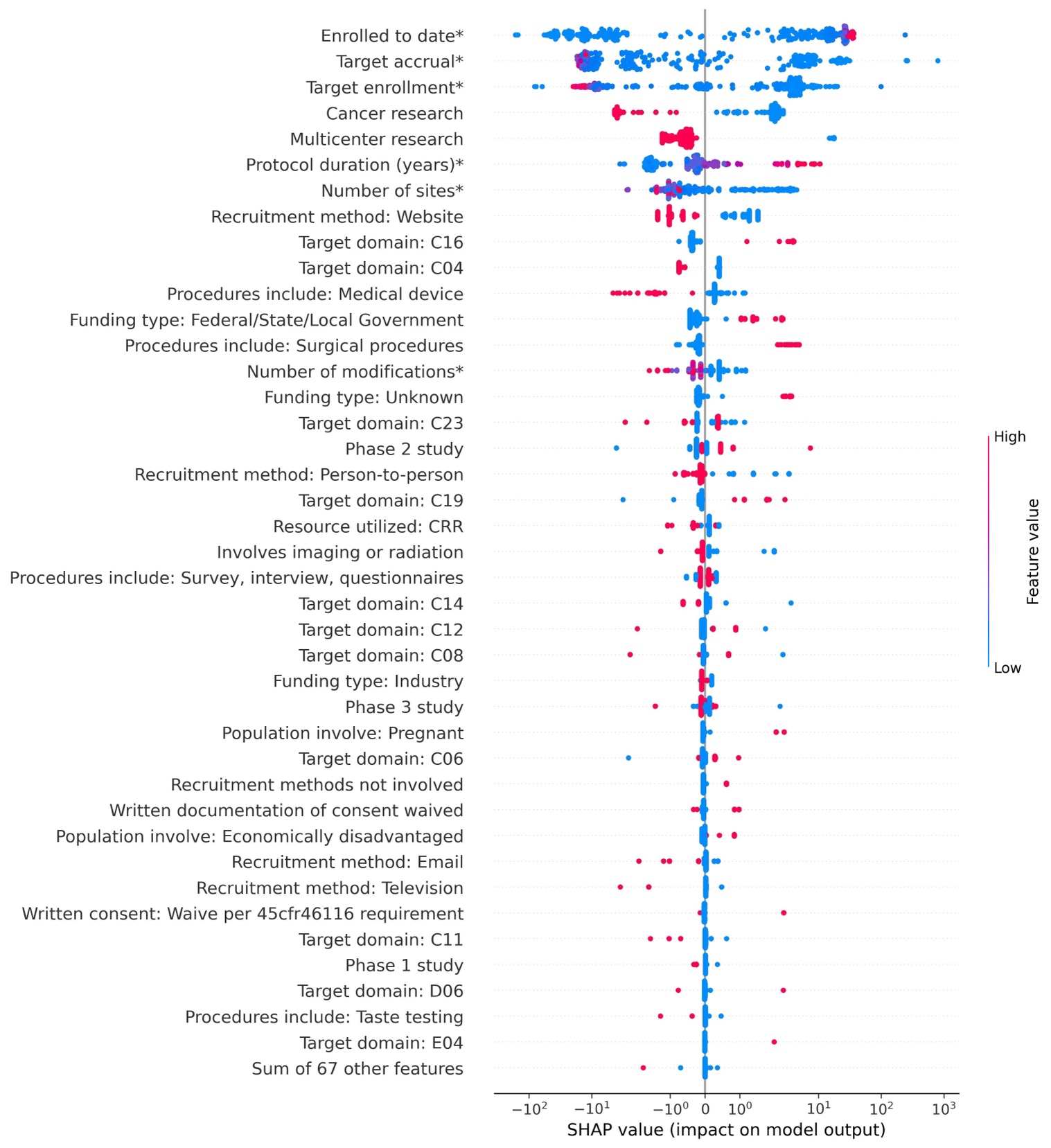


*Note*: * Features are continuous variables, whereas the others are binary variables. The SHAP values have been log scaled. C16: Congenital, Hereditary, and Neonatal Diseases and Abnormalities. C04: Neoplasms. C23: Pathological Conditions, Signs and Symptoms. C19: Endocrine System Diseases. C14: Cardiovascular Diseases. C12: Male Urogenital Diseases. C08: Respiratory Tract Diseases. C06: Digestive System Diseases. C11: Eye Diseases. D06: Hormones, Hormone Substitutes, and Hormone Antagon. E04: Surgical Procedures, Operative.

**Table S3.** Comparison of the Top 30 Most Important Features between the Best (Accrual Percentage $\geq$ 90%, n = 64) and Worst (Accrual Percentage $\leq$ 10%, n = 60) Recruitment Group based on 393 Trials.

| **Feature** | **Worst recruitment group**  **Count (%) / Median (Q1, Q3)** | **Best recruitment group**  **Count (%) / Median (Q1, Q3)** | **Corrected p-value** |
| --- | --- | --- | --- |
| Enrolled to date* | 1.0, (0.75, 3.5) | 11.0, (4.0, 40.25) | 1.53E-23^†^ |
| Target enrollment* | 15.0, (10.0, 40.0) | 15.0, (7.75, 60.0) | 9.84E-42^†^ |
| Target accrual* | 10.0, (9.5, 25.0) | 10.0, (4.0, 33.5) | 3.99E-41^†^ |
| Recruitment method: Website | 44, 0.73 | 15, 0.23 | 1.08E-06^†^ |
| Cancer research | 32, 0.53 | 6, 0.09 | 2.36E-06^†^ |
| Multicenter research | 60, 1.0 | 54, 0.84 | 4.17E-02^†^ |
| Protocol duration (years) * | 4.0, (2.0, 5.0) | 5.5, (3.0, 9.0) | 2.98E-40^†^ |
| Funding type: Industry | 44, 0.73 | 35, 0.55 | 1.00E+00 |
| Number of sites* | 74.5, (39.75, 138.25) | 36.5, (2.75, 88.75) | 2.10E-38^†^ |
| Procedures include: Medical device | 13, 0.22 | 6, 0.09 | 1.00E+00 |
| Funding type: Federal/State/Local Government | 4, 0.07 | 16, 0.25 | 2.02E-01 |
| Target domain: C16 | 2, 0.03 | 11, 0.17 | 5.06E-01 |
| Target domain: C04 | 32, 0.53 | 9, 0.14 | 1.05E-04^†^ |
| Procedures include: Surgical procedures | 5, 0.08 | 4, 0.06 | 1.00E+00 |
| Written documentation of consent waived | 1, 0.02 | 10, 0.16 | 2.66E-01 |
| Funding type: Unknown | 3, 0.05 | 10, 0.16 | 1.00E+00 |
| Involves compensation | 25, 0.42 | 44, 0.69 | 1.10E-01 |
| Target domain: C10 | 8, 0.13 | 17, 0.27 | 1.00E+00 |
| No resources utilized | 22, 0.37 | 43, 0.67 | 3.35E-02^†^ |
| Target domain: C06 | 10, 0.17 | 14, 0.22 | 1.00E+00 |
| Population involve: Economically disadvantaged | 4, 0.07 | 11, 0.17 | 1.00E+00 |
| Population involve: Pregnant | 0, 0.0 | 6, 0.09 | 8.40E-01 |
| Target domain: C19 | 3, 0.05 | 7, 0.11 | 1.00E+00 |
| Target domain: C15 | 6, 0.1 | 2, 0.03 | 1.00E+00 |
| Phase 2 study | 22, 0.37 | 23, 0.36 | 1.00E+00 |
| Involves imaging or radiation | 42, 0.7 | 30, 0.47 | 3.32E-01 |
| Written consent: non-English language not expected | 18, 0.3 | 26, 0.41 | 1.00E+00 |
| Recruitment methods not involved | 5, 0.08 | 9, 0.14 | 1.00E+00 |
| Number of modifications* | 1.0, (0.0, 2.0) | 0.0, (0.0, 1.0) | 1.00E+00 |
| Phase 3 study | 31, 0.52 | 31, 0.48 | 1.00E+00 |

*Note*. Mann–Whitney U and Fisher's Exact tests with Bonferroni correction were used for continuous and binary variables, respectively. The descriptive statistics of each feature for these two subgroups are also listed. * Features are continuous variables where the median, the first quantile (Q1), and the third quantile (Q3) were calculated, whereas the others are binary variables where the count and the percentage were calculated. ^†^ Corrected p-value <.05. C16: Congenital, hereditary, and neonatal diseases and abnormalities. CO4: Neoplasms. C10: Nervous System Diseases. C06: Digestive System Diseases. C19: Endocrine System Diseases. C15: Hemic and lymphatic diseases. SD: Standard deviation.

**Table S4.** Comparison of the Top 30 Most Important Features between the Best (Accrual Percentage $\geq$ 80%, n = 87) and Worst (Accrual Percentage $\leq$ 20%, n = 110) Recruitment Group based on 393 Trials.

| **Feature** | **Worst recruitment group**  **Count (%) / Median (Q1, Q3)** | **Best recruitment group**  **Count (%) / Median (Q1, Q3)** | **Corrected p-value** |
| --- | --- | --- | --- |
| Enrolled to date* | 2.0, (1.0, 5.0) | 11.0, (5.0, 43.5) | 4.10E-46^†^ |
| Target enrollment* | 15.0, (10.0, 40.0) | 15.0, (8.0, 60.0) | 1.55E-66^†^ |
| Target accrual* | 10.0, (8.0, 25.0) | 10.0, (5.0, 35.0) | 5.22E-66^†^ |
| Recruitment method: Website | 75, 0.68 | 25, 0.29 | 1.14E-06^†^ |
| Cancer research | 56, 0.51 | 8, 0.09 | 4.11E-09^†^ |
| Multicenter research | 110, 1.0 | 74, 0.85 | 4.26E-04^†^ |
| Protocol duration (years)* | 4.0, (2.25, 5.75) | 5.0, (3.0, 8.0) | 3.30E-64^†^ |
| Funding type: Industry | 81, 0.74 | 52, 0.6 | 1.00E+00 |
| Number of sites* | 77.5, (39.25, 154.75) | 36.0, (7.0, 90.0) | 5.14E-62^†^ |
| Procedures include: Medical device | 23, 0.21 | 12, 0.14 | 1.00E+00 |
| Funding type: Federal/State/Local Government | 8, 0.07 | 20, 0.23 | 6.05E-02 |
| Target domain: C16 | 3, 0.03 | 16, 0.18 | 1.07E-02^†^ |
| Target domain: C04 | 53, 0.48 | 11, 0.13 | 3.25E-06^†^ |
| Procedures include: Surgical procedures | 7, 0.06 | 6, 0.07 | 1.00E+00 |
| Written documentation of consent waived | 2, 0.02 | 11, 0.13 | 8.94E-02 |
| Funding type: Unknown | 4, 0.04 | 12, 0.14 | 4.74E-01 |
| Involves compensation | 40, 0.36 | 62, 0.71 | 5.12E-05^†^ |
| Target domain: C10 | 20, 0.18 | 23, 0.26 | 1.00E+00 |
| No resources utilized | 44, 0.4 | 58, 0.67 | 9.24E-03^†^ |
| Target domain: C06 | 14, 0.13 | 20, 0.23 | 1.00E+00 |
| Population involve: Economically disadvantaged | 8, 0.07 | 13, 0.15 | 1.00E+00 |
| Population involve: Pregnant | 1, 0.01 | 6, 0.07 | 1.00E+00 |
| Target domain: C19 | 5, 0.05 | 9, 0.1 | 1.00E+00 |
| Target domain: C15 | 15, 0.14 | 4, 0.05 | 1.00E+00 |
| Phase 2 study | 39, 0.35 | 31, 0.36 | 1.00E+00 |
| Involves imaging or radiation | 78, 0.71 | 41, 0.47 | 2.35E-02^†^ |
| Written consent: non-English language not expected | 35, 0.32 | 32, 0.37 | 1.00E+00 |
| Recruitment methods not involved | 7, 0.06 | 10, 0.11 | 1.00E+00 |
| Number of modifications* | 1.0, (0.0, 2.0) | 0.0, (0.0, 1.0) | 9.21E-02 |
| Phase 3 study | 56, 0.51 | 42, 0.48 | 1.00E+00 |

*Note*. Mann–Whitney U and Fisher's Exact tests with Bonferroni correction were used for continuous and binary variables, respectively. The descriptive statistics of each feature for these two subgroups are also listed. * Features are continuous variables where the median, the first quantile (Q1), and the third quantile (Q3) were calculated, whereas the others are binary variables where the count and the percentage were calculated. ^†^ Corrected p-value <.05. C16: Congenital, hereditary, and neonatal diseases and abnormalities. CO4: Neoplasms. C10: Nervous System Diseases. C06: Digestive System Diseases. C19: Endocrine System Diseases. C15: Hemic and lymphatic diseases. SD: Standard deviation.

**Table S5.** Comparison of the Top 30 Most Important Features between the Best (Accrual Percentage $\geq$ 70%, n = 108) and Worst (Accrual Percentage $\leq$ 30%, n = 155) Recruitment Group based on 393 Trials.

| **Feature** | **Worst recruitment group**  **Count (%) / Median (Q1, Q3)** | | **Best recruitment group**  **Count (%) / Median (Q1, Q3)** | **Corrected p-value** |
| --- | --- | --- | --- | --- |
| Enrolled to date* | 3.0, (1.0, 8.0) | 13.0, (6.0, 37.0) | | 1.98E-68^†^ |
| Target enrollment* | 16.0, (10.0, 40.0) | 17.0, (10.0, 60.0) | | 5.10E-89^†^ |
| Target accrual* | 10.0, (8.0, 25.0) | 10.0, (6.0, 33.5) | | 1.60E-88^†^ |
| Recruitment method: Website | 106, 0.68 | 34, 0.31 | | 1.48E-07^†^ |
| Cancer research | 78, 0.5 | 13, 0.12 | | 1.30E-09^†^ |
| Multicenter research | 155, 1.0 | 94, 0.87 | | 6.86E-05^†^ |
| Protocol duration (years)* | 4.0, (3.0, 6.0) | 5.0, (3.0, 7.0) | | 2.40E-86^†^ |
| Funding type: Industry | 109, 0.7 | 64, 0.59 | | 1.00E+00 |
| Number of sites* | 81.0, (39.5, 156.0) | 31.0, (9.5, 88.25) | | 5.48E-84^†^ |
| Procedures include: Medical device | 32, 0.21 | 13, 0.12 | | 1.00E+00 |
| Funding type: Federal/State/Local Government | 13, 0.08 | 29, 0.27 | | 3.58E-03^†^ |
| Target domain: C16 | 6, 0.04 | 18, 0.17 | | 2.20E-02^†^ |
| Target domain: C04 | 76, 0.49 | 16, 0.15 | | 1.50E-07^†^ |
| Procedures include: Surgical procedures | 9, 0.06 | 8, 0.07 | | 1.00E+00 |
| Written documentation of consent waived | 2, 0.01 | 11, 0.1 | | 6.51E-02 |
| Funding type: Unknown | 5, 0.03 | 13, 0.12 | | 3.29E-01 |
| Involves compensation | 59, 0.38 | 75, 0.69 | | 2.45E-05^†^ |
| Target domain: C10 | 30, 0.19 | 31, 0.29 | | 1.00E+00 |
| No resources utilized | 62, 0.4 | 66, 0.61 | | 3.21E-02^†^ |
| Target domain: C06 | 18, 0.12 | 22, 0.2 | | 1.00E+00 |
| Population involve: Economically disadvantaged | 10, 0.06 | 14, 0.13 | | 1.00E+00 |
| Population involve: Pregnant | 1, 0.01 | 7, 0.06 | | 2.75E-01 |
| Target domain: C19 | 6, 0.04 | 10, 0.09 | | 1.00E+00 |
| Target domain: C15 | 22, 0.14 | 6, 0.06 | | 7.93E-01 |
| Phase 2 study | 52, 0.34 | 38, 0.35 | | 1.00E+00 |
| Involves imaging or radiation | 111, 0.72 | 54, 0.5 | | 1.36E-02^†^ |
| Written consent: non-English language not expected | 58, 0.37 | 43, 0.4 | | 1.00E+00 |
| Recruitment methods not involved | 8, 0.05 | 11, 0.1 | | 1.00E+00 |
| Number of modifications* | 1.0, (0.0, 2.0) | 0.0, (0.0, 1.0) | | 3.22E-04^†^ |
| Phase 3 study | 82, 0.53 | 55, 0.51 | | 1.00E+00 |

*Note*. Mann–Whitney U and Fisher's Exact tests with Bonferroni correction were used for continuous and binary variables, respectively. The descriptive statistics of each feature for these two subgroups are also listed. * Features are continuous variables where the median, the first quantile (Q1), and the third quantile (Q3) were calculated, whereas the others are binary variables where the count and the percentage were calculated. ^†^ Corrected p-value <.05. C16: Congenital, hereditary, and neonatal diseases and abnormalities. CO4: Neoplasms. C10: Nervous System Diseases. C06: Digestive System Diseases. C19: Endocrine System Diseases. C15: Hemic and lymphatic diseases. SD: Standard deviation.

**Table S6.** Features with a Significant Difference (Corrected P-value <.05) between the Worst and Best Recruitment Group under different cut-offs (i.e., $\leq$ 10% vs. $\geq$ 90%, $\leq$ 20% vs. $\geq$ 80%, and $\leq$ 30% vs. $\geq$ 70%) based on 400 Trials (including 7 Trials with Accrual Percentage >100%) among the Top 30 Most Important Features.

| **Cut-off** | **Features** | **Worst recruitment group**  **Count (%) / Median (Q1, Q3)** | **Best recruitment group**  **Count (%) / Median (Q1, Q3)** | **Corrected p-value** |
| --- | --- | --- | --- | --- |
| $\leq$ 10 (n=60) vs. $\geq$ 90 (n=71) | Enrolled to date* | 1.0, (0.75, 3.5) | 14.0, (5.0, 50.0) | 2.47E-25 |
|  | Target accrual* | 10.0, (9.5, 25.0) | 10.0, (4.5, 39.0) | 3.68E-43 |
|  | Target enrollment* | 15.0, (10.0, 40.0) | 18.0, (8.0, 61.5) | 4.12E-44 |
|  | Cancer research | 32, 0.53 | 7, 0.1 | 2.40E-06 |
|  | Multicenter research | 60, 1.0 | 60, 0.85 | 2.75E-02 |
|  | Protocol duration (years)* | 4.0, (2.0, 5.0) | 5.0, (3.5, 8.5) | 1.46E-42 |
|  | Number of sites* | 74.5, (39.75, 138.25) | 26.0, (3.0, 84.5) | 2.51E-40 |
|  | Recruitment method: Website | 44, 0.73 | 19, 0.27 | 3.13E-06 |
|  | Target domain: C04 | 32, 0.53 | 11, 0.15 | 1.62E-04 |
| $\leq$ 20 (n=110) vs. $\geq$ 80 (n=94) | Enrolled to date* | 2.0, (1.0, 5.0) | 13.0, (6.0, 50.0) | 6.85E-48 |
|  | Target accrual* | 10.0, (8.0, 25.0) | 10.0, (5.0, 38.0) | 5.11E-68 |
|  | Target enrollment* | 15.0, (10.0, 40.0) | 15.0, (10.0, 62.25) | 7.70E-69 |
|  | Cancer research | 56, 0.51 | 9, 0.1 | 2.56E-09 |
|  | Multicenter research | 110, 1.0 | 80, 0.85 | 3.33E-04 |
|  | Protocol duration (years)* | 4.0, (2.25, 5.75) | 5.0, (3.0, 7.75) | 2.02E-66 |
|  | Number of sites* | 77.5, (39.25, 154.75) | 29.5, (6.5, 88.75) | 7.00E-64 |
|  | Recruitment method: Website | 75, 0.68 | 29, 0.31 | 4.41E-06 |
|  | Target domain: C16 | 3, 0.03 | 17, 0.18 | 7.45E-03 |
|  | Target domain: C04 | 53, 0.48 | 13, 0.14 | 3.40E-06 |
|  | Involves imaging or radiation | 78, 0.71 | 44, 0.47 | 1.73E-02 |
| $\leq$ 30 (n=155) vs. $\geq$ 70 (n=115) | Enrolled to date* | 3.0, (1.0, 8.0) | 15.0, (7.0, 38.0) | 2.90E-70 |
|  | Target accrual* | 10.0, (8.0, 25.0) | 10.0, (6.0, 38.0) | 1.61E-90 |
|  | Target enrollment* | 16.0, (10.0, 40.0) | 20.0, (10.0, 60.0) | 2.74E-91 |
|  | Cancer research | 78, 0.5 | 14, 0.12 | 5.03E-10 |
|  | Multicenter research | 155, 1.0 | 100, 0.87 | 4.73E-05 |
|  | Protocol duration (years)* | 4.0, (3.0, 6.0) | 5.0, (3.0, 7.0) | 1.57E-88 |
|  | Number of sites* | 81.0, (39.5, 156.0) | 30.0, (8.0, 84.5) | 7.62E-86 |
|  | Recruitment method: Website | 106, 0.68 | 38, 0.33 | 3.03E-07 |
|  | Target domain: C16 | 6, 0.04 | 19, 0.17 | 1.54E-02 |
|  | Target domain: C04 | 76, 0.49 | 18, 0.16 | 2.46E-07 |
|  | Funding type: Federal/State/Local Government | 13, 0.08 | 30, 0.26 | 4.80E-03 |
|  | Number of modifications* | 1.0, (0.0, 2.0) | 0.0, (0.0, 1.0) | 1.80E-03 |
|  | Involves imaging or radiation | 111, 0.72 | 57, 0.5 | 1.06E-02 |

*Note*. Mann–Whitney U and Fisher's Exact tests with Bonferroni correction were used for continuous and binary variables, respectively. The descriptive statistics of each feature for these two subgroups are also listed. *Features are continuous variables where the median, the first quantile (Q1), and the third quantile (Q3) were calculated, whereas the others are binary variables where the count and the percentage were calculated. CO4: Neoplasms. C16: Congenital, hereditary, and neonatal diseases and abnormalities.

**Table S7.** Comparison of the Top 30 Most Important Features between the Best (Accrual Percentage $\geq$ 90%, n = 71) and Worst (Accrual Percentage $\leq$ 10%, n = 60) Recruitment Group based on 400 Trials (including 7 Trials with Accrual Percentage >100%).

| **Feature** | **Worst recruitment group Count (%) / Median (Q1, Q3)** | **Best recruitment group Count (%) / Median (Q1, Q3)** | **Corrected p-value** |
| --- | --- | --- | --- |
| Enrolled to date* | 1.0, (0.75, 3.5) | 14.0, (5.0, 50.0) | 2.47E-25^†^ |
| Target accrual* | 10.0, (9.5, 25.0) | 10.0, (4.5, 39.0) | 3.68E-43^†^ |
| Target enrollment* | 15.0, (10.0, 40.0) | 18.0, (8.0, 61.5) | 4.12E-44^†^ |
| Cancer research | 32, 0.53 | 7, 0.1 | 2.40E-06^†^ |
| Multicenter research | 60, 1.0 | 60, 0.85 | 2.75E-02^†^ |
| Protocol duration (years)* | 4.0, (2.0, 5.0) | 5.0, (3.5, 8.5) | 1.46E-42^†^ |
| Number of sites* | 74.5, (39.75, 138.25) | 26.0, (3.0, 84.5) | 2.51E-40^†^ |
| Recruitment method: Website | 44, 0.73 | 19, 0.27 | 3.13E-06^†^ |
| Target domain: C16 | 2, 0.03 | 12, 0.17 | 6.11E-01 |
| Target domain: C04 | 32, 0.53 | 11, 0.15 | 1.62E-04^†^ |
| Procedures include: Medical device | 13, 0.22 | 8, 0.11 | 1.00E+00 |
| Funding type: Federal/State/Local Government | 4, 0.07 | 17, 0.24 | 2.49E-01 |
| Procedures include: Surgical procedures | 5, 0.08 | 5, 0.07 | 1.00E+00 |
| Number of modifications* | 1.0, (0.0, 2.0) | 0.0, (0.0, 1.0) | 1.00E+00 |
| Funding type: Unknown | 3, 0.05 | 11, 0.15 | 1.00E+00 |
| Target domain: C23 | 16, 0.27 | 29, 0.41 | 1.00E+00 |
| Phase 2 study | 22, 0.37 | 25, 0.35 | 1.00E+00 |
| Recruitment method: Person-to-person | 55, 0.92 | 60, 0.85 | 1.00E+00 |
| Target domain: C19 | 3, 0.05 | 8, 0.11 | 1.00E+00 |
| Resource utilized: CRR | 16, 0.27 | 20, 0.28 | 1.00E+00 |
| Involves imaging or radiation | 42, 0.7 | 33, 0.46 | 2.41E-01 |
| Procedures include: Survey, interview, questionnaires | 40, 0.67 | 56, 0.79 | 1.00E+00 |
| Target domain: C14 | 11, 0.18 | 12, 0.17 | 1.00E+00 |
| Target domain: C12 | 3, 0.05 | 8, 0.11 | 1.00E+00 |
| Target domain: C08 | 5, 0.08 | 11, 0.15 | 1.00E+00 |
| Funding type: Industry | 44, 0.73 | 40, 0.56 | 1.00E+00 |
| Phase 3 study | 31, 0.52 | 33, 0.46 | 1.00E+00 |
| Population involve: Pregnant | 0, 0.0 | 6, 0.08 | 9.28E-01 |
| Target domain: C06 | 10, 0.17 | 16, 0.23 | 1.00E+00 |
| Recruitment methods not involved | 5, 0.08 | 10, 0.14 | 1.00E+00 |

*Note*. Mann–Whitney U and Fisher's Exact tests with Bonferroni correction were used for continuous and binary variables, respectively. The descriptive statistics of each feature for these two subgroups are also listed. * Features are continuous variables where the median, the first quantile (Q1), and the third quantile (Q3) were calculated, whereas the others are binary variables where the count and the percentage were calculated. ^†^ Corrected p-value <.05. C16: Congenital, Hereditary, and Neonatal Diseases and Abnormalities. C04: Neoplasms. C23: Pathological Conditions, Signs and Symptoms. C19: Endocrine System Diseases. C14: Cardiovascular Diseases. C12: Male Urogenital Diseases. C08: Respiratory Tract Diseases. C06: Digestive System Diseases.

**Table S8.** Comparison of the Top 30 Most Important Features between the Best (Accrual Percentage $\geq$ 80%, n = 94) and Worst (Accrual Percentage $\leq$ 20%, n = 110) Recruitment Group based on 400 Trials (including 7 Trials with Accrual Percentage >100%).

| **Feature** | **Worst recruitment group Count (%) / Median (Q1, Q3)** | **Best recruitment group Count (%) / Median (Q1, Q3)** | **Corrected p-value** |
| --- | --- | --- | --- |
| Enrolled to date* | 2.0, (1.0, 5.0) | 13.0, (6.0, 50.0) | 6.85E-48^†^ |
| Target accrual* | 10.0, (8.0, 25.0) | 10.0, (5.0, 38.0) | 5.11E-68^†^ |
| Target enrollment* | 15.0, (10.0, 40.0) | 15.0, (10.0, 62.25) | 7.70E-69^†^ |
| Cancer research | 56, 0.51 | 9, 0.1 | 2.56E-09^†^ |
| Multicenter research | 110, 1.0 | 80, 0.85 | 3.33E-04^†^ |
| Protocol duration (years)* | 4.0, (2.25, 5.75) | 5.0, (3.0, 7.75) | 2.02E-66^†^ |
| Number of sites* | 77.5, (39.25, 154.75) | 29.5, (6.5, 88.75) | 7.00E-64^†^ |
| Recruitment method: Website | 75, 0.68 | 29, 0.31 | 4.41E-06^†^ |
| Target domain: C16 | 3, 0.03 | 17, 0.18 | 7.45E-03^†^ |
| Target domain: C04 | 53, 0.48 | 13, 0.14 | 3.40E-06^†^ |
| Procedures include: Medical device | 23, 0.21 | 14, 0.15 | 1.00E+00 |
| Funding type: Federal/State/Local Government | 8, 0.07 | 21, 0.22 | 7.40E-02 |
| Procedures include: Surgical procedures | 7, 0.06 | 7, 0.07 | 1.00E+00 |
| Number of modifications* | 1.0, (0.0, 2.0) | 0.0, (0.0, 1.0) | 3.49E-01 |
| Funding type: Unknown | 4, 0.04 | 13, 0.14 | 3.18E-01 |
| Target domain: C23 | 38, 0.35 | 40, 0.43 | 1.00E+00 |
| Phase 2 study | 39, 0.35 | 33, 0.35 | 1.00E+00 |
| Recruitment method: Person-to-person | 102, 0.93 | 82, 0.87 | 1.00E+00 |
| Target domain: C19 | 5, 0.05 | 10, 0.11 | 1.00E+00 |
| Resource utilized: CRR | 27, 0.25 | 26, 0.28 | 1.00E+00 |
| Involves imaging or radiation | 78, 0.71 | 44, 0.47 | 1.73E-02^†^ |
| Procedures include: Survey, interview, questionnaires | 75, 0.68 | 74, 0.79 | 1.00E+00 |
| Target domain: C14 | 22, 0.2 | 18, 0.19 | 1.00E+00 |
| Target domain: C12 | 10, 0.09 | 11, 0.12 | 1.00E+00 |
| Target domain: C08 | 9, 0.08 | 13, 0.14 | 1.00E+00 |
| Funding type: Industry | 81, 0.74 | 57, 0.61 | 1.00E+00 |
| Phase 3 study | 56, 0.51 | 44, 0.47 | 1.00E+00 |
| Population involve: Pregnant | 1, 0.01 | 6, 0.06 | 1.00E+00 |
| Target domain: C06 | 14, 0.13 | 22, 0.23 | 1.00E+00 |
| Recruitment methods not involved | 7, 0.06 | 11, 0.12 | 1.00E+00 |

*Note*. Mann–Whitney U and Fisher's Exact tests with Bonferroni correction were used for continuous and binary variables, respectively. The descriptive statistics of each feature for these two subgroups are also listed. * Features are continuous variables where the median, the first quantile (Q1), and the third quantile (Q3) were calculated, whereas the others are binary variables where the count and the percentage were calculated. ^†^ Corrected p-value <.05. C16: Congenital, Hereditary, and Neonatal Diseases and Abnormalities. C04: Neoplasms. C23: Pathological Conditions, Signs and Symptoms. C19: Endocrine System Diseases. C14: Cardiovascular Diseases. C12: Male Urogenital Diseases. C08: Respiratory Tract Diseases. C06: Digestive System Diseases.

**Table S9.** Comparison of the Top 30 Most Important Features between the Best (Accrual Percentage $\geq$ 70%, n = 115) and Worst (Accrual Percentage $\leq$ 30%, n = 155) Recruitment Group based on 400 Trials (including 7 Trials with Accrual Percentage >100%).

| **Feature** | **Worst recruitment group**  **Count (%) / Median (Q1, Q3)** | **Best recruitment group**  **Count (%) / Median (Q1, Q3)** | **Corrected p-value** |
| --- | --- | --- | --- |
| Enrolled to date* | 3.0, (1.0, 8.0) | 15.0, (7.0, 38.0) | 2.90E-70^†^ |
| Target accrual* | 10.0, (8.0, 25.0) | 10.0, (6.0, 38.0) | 1.61E-90^†^ |
| Target enrollment* | 16.0, (10.0, 40.0) | 20.0, (10.0, 60.0) | 2.74E-91^†^ |
| Cancer research | 78, 0.5 | 14, 0.12 | 5.03E-10^†^ |
| Multicenter research | 155, 1.0 | 100, 0.87 | 4.73E-05^†^ |
| Protocol duration (years)* | 4.0, (3.0, 6.0) | 5.0, (3.0, 7.0) | 1.57E-88^†^ |
| Number of sites* | 81.0, (39.5, 156.0) | 30.0, (8.0, 84.5) | 7.62E-86^†^ |
| Recruitment method: Website | 106, 0.68 | 38, 0.33 | 3.03E-07^†^ |
| Target domain: C16 | 6, 0.04 | 19, 0.17 | 1.54E-02^†^ |
| Target domain: C04 | 76, 0.49 | 18, 0.16 | 2.46E-07^†^ |
| Procedures include: Medical device | 32, 0.21 | 15, 0.13 | 1.00E+00 |
| Funding type: Federal/State/Local Government | 13, 0.08 | 30, 0.26 | 4.80E-03^†^ |
| Procedures include: Surgical procedures | 9, 0.06 | 9, 0.08 | 1.00E+00 |
| Number of modifications* | 1.0, (0.0, 2.0) | 0.0, (0.0, 1.0) | 1.80E-03^†^ |
| Funding type: Unknown | 5, 0.03 | 14, 0.12 | 2.05E-01 |
| Target domain: C23 | 53, 0.34 | 48, 0.42 | 1.00E+00 |
| Phase 2 study | 52, 0.34 | 40, 0.35 | 1.00E+00 |
| Recruitment method: Person-to-person | 146, 0.94 | 103, 0.9 | 1.00E+00 |
| Target domain: C19 | 6, 0.04 | 11, 0.1 | 1.00E+00 |
| Resource utilized: CRR | 38, 0.25 | 35, 0.3 | 1.00E+00 |
| Involves imaging or radiation | 111, 0.72 | 57, 0.5 | 1.06E-02^†^ |
| Procedures include: Survey, interview, questionnaires | 107, 0.69 | 88, 0.77 | 1.00E+00 |
| Target domain: C14 | 30, 0.19 | 20, 0.17 | 1.00E+00 |
| Target domain: C12 | 12, 0.08 | 14, 0.12 | 1.00E+00 |
| Target domain: C08 | 10, 0.06 | 16, 0.14 | 1.00E+00 |
| Funding type: Industry | 109, 0.7 | 69, 0.6 | 1.00E+00 |
| Phase 3 study | 82, 0.53 | 57, 0.5 | 1.00E+00 |
| Population involve: Pregnant | 1, 0.01 | 7, 0.06 | 3.51E-01 |
| Target domain: C06 | 18, 0.12 | 24, 0.21 | 1.00E+00 |
| Recruitment methods not involved | 8, 0.05 | 12, 0.1 | 1.00E+00 |

*Note*. Mann–Whitney U and Fisher's Exact tests with Bonferroni correction were used for continuous and binary variables, respectively. The descriptive statistics of each feature for these two subgroups are also listed. * Features are continuous variables where the median, the first quantile (Q1), and the third quantile (Q3) were calculated, whereas the others are binary variables where the count and the percentage were calculated. ^†^ Corrected p-value <.05. C16: Congenital, Hereditary, and Neonatal Diseases and Abnormalities. C04: Neoplasms. C23: Pathological Conditions, Signs and Symptoms. C19: Endocrine System Diseases. C14: Cardiovascular Diseases. C12: Male Urogenital Diseases. C08: Respiratory Tract Diseases. C06: Digestive System Diseases.

**REFERENCES**

1. Bellomo R, Warrillow SJ, Reade MC. Why we should be wary of single-center trials. *Critical Care Medicine*. 2009; **37**(12):3114-3119. doi:10.1097/CCM.0b013e3181bc7bd5

2. Stein MA, Shaffer M, Echo‐Hawk A, Smith J, Stapleton A, Melvin A. Research START: a multimethod study of barriers and accelerators of recruiting research participants. *Clinical and Translational Science*. 2015;8(6):647-654. doi:10.1111/cts.12351

3. Hildebrand JA, Billimek J, Olshansky EF, et al. Facilitators and barriers to research participation: Perspectives of Latinos with type 2 diabetes. *European Journal of Cardiovascular Nursing*. 2018; **17**(8):737-741. doi:10.1177/1474515118780895

4. George S, Duran N, Norris K. A systematic review of barriers and facilitators to minority research participation among African Americans, Latinos, Asian Americans, and Pacific Islanders. *American Journal of Public Health*. 2014; **104**(2):e16-31. doi:10.2105/ajph.2013.301706

5. Treweek S, Pitkethly M, Cook J, et al. Strategies to improve recruitment to randomised trials. *Cochrane Database of Systematic Reviews*. 2018; **2**(2): MR000013. doi:10.1002/14651858.MR000013.pub6

6. Quay TA, Frimer L, Janssen PA, et al. Barriers and facilitators to recruitment of South Asians to health research: A scoping review. *BMJ Open*. 2017; **7**(5):e014889. doi:10.1136/bmjopen-2016-014889 Quay TA, Frimer L, Janssen PA, et al. Barriers and facilitators to recruitment of South Asians to health research: A scoping review. *BMJ Open*. 2017; **7**(5):e014889. doi:10.1136/bmjopen-2016-014889

7. Idnay B, Dreisbach C, Weng C, Schnall R. A systematic review on natural language processing systems for eligibility prescreening in clinical research. *Journal of the American Medical Informatics Association*. 2021; **29**(1):197-206. doi:10.1093/jamia/ocab228

8. Palac HL, Alam N, Kaiser SM, et al. A Practical Do-It-Yourself Recruitment Framework for Concurrent eHealth Clinical Trials: Simple Architecture (Part 1). *Journal of Medical Internet research*. 2018 ;**20**(11):e11049-e11049. doi:10.2196/11049

9. Hoffman KA, Baker R, Kunkel LE, et al. Barriers and facilitators to recruitment and enrollment of HIV-infected individuals with opioid use disorder in a clinical trial. *BMC Health Services Research*. 2019; **19**(1):862. doi:10.1186/s12913-019-4721-x

10. Howcutt SJ, Barnett AL, Barbosa-Boucas S, Smith LA. Research recruitment: A marketing framework to improve sample representativeness in health research. *Journal of Advanced Nursing*. 2018; **74**(4):968-975. doi:10.1111/jan.13490

11. Fogel DB. Factors associated with clinical trials that fail and opportunities for improving the likelihood of success: A review. *Contemporary Clinical Trials Communications*. 2018; **11**:156-164. doi:10.1016/j.conctc.2018.08.001

12. Haddad RI, Chan AT, Vermorken JB. Barriers to clinical trial recruitment in head and neck cancer. *Oral Oncology*. 2015; **51**(3):203-11. doi:10.1016/j.oraloncology.2014.12.007

13. Hay M, Thomas DW, Craighead JL, et al. Clinical development success rates for investigational drugs. *Nature Biotechnology*. 2014; **32**(1):40-51. doi:10.1038/nbt.2786

14. Pinato DJ, Stavraka C, Flynn MJ, et al. An inflammation based score can optimize the selection of patients with advanced cancer considered for early phase clinical trials. *PloS One*. 2014; **9**(1):e83279. doi:10.1371/journal.pone.0083279

15. Weber JS, Levit LA, Adamson PC, et al. American Society of Clinical Oncology policy statement update: The critical role of phase I trials in cancer research and treatment. *Journal of Clinical Oncology*. 2015; **33**(3):278-84. doi:10.1200/jco.2014.58.2635
